# Heterologous biosynthesis of cotylenol and concise synthesis of fusicoccane diterpenoids

**DOI:** 10.3762/bjoc.21.111

**Published:** 2025-07-21

**Authors:** Ye Yuan, Zhenhua Guan, Xue-Jie Zhang, Nanyu Yao, Wenling Yuan, Yonghui Zhang, Ying Ye, Zheng Xiang

**Affiliations:** 1 State Key Laboratory of Chemical Oncogenomics, Shenzhen Key Laboratory of Chemical Genomics, School of Chemical Biology and Biotechnology, Peking University Shenzhen Graduate School, Shenzhen 518055, P. R. China; 2 Hubei Key Laboratory of Natural Medicinal Chemistry and Resource Evaluation, School of Pharmacy, Tongji Medical College, Huazhong University of Science and Technology, Wuhan 430030, P. R. China; 3 Institute of Chemical Biology, Shenzhen Bay Laboratory, Shenzhen 518132, P. R. China

**Keywords:** cotylenol, fusicoccane diterpenoids, heterologous biosynthesis, P450 oxidation, synthesis

## Abstract

A novel strategy for the synthesis of fusicoccane diterpenoids is reported. By harnessing the biosynthetic pathways of brassicicenes and fusicoccins, cotylenol was produced in an engineered *Aspergillus oryzae* strain. We further achieved the concise synthesis of three fusicoccane diterpenoids, including alterbrassicicene E and brassicicenes A and R in 4 or 5 chemical steps from brassicicene I. This strategy lays the foundation for the preparation of fusicoccane diterpenoids and their analogues for biological studies.

## Introduction

**Figure 1:**
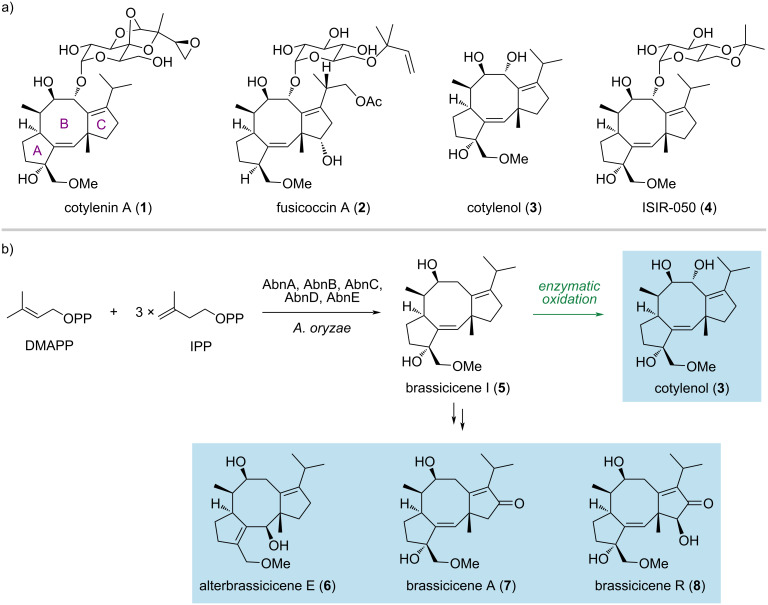
Selected fusicoccane diterpenoids and overview of this study. (a) Representative members of the fusicoccane diterpenoid family. (b) This work: heterologous biosynthesis of cotylenol (**3**) in an engineered *Aspergillus oryzae* strain and concise synthesis of fusicoccane diterpenoids.

Fusicoccanes are a family of 5-8-5 tricyclic diterpenoid natural products that are produced by bacteria, fungi, algae, and plants (Figure 1a) [1–7]. Fusicoccanes possess a broad range of biological activities, including anticancer, anti-inflammatory, antimicrobial, antiparasitic, and plant growth regulating activities. For instance, cotylenin A (**1**) and fusicoccin A (**2**) function as molecular glues to stabilize the interactions between 14-3-3 proteins and their binding partners in plant and animal cells [8–12]. It has been reported that cotylenin A and its aglycone, cotylenol (**3**), induce differentiation in murine and human myeloid leukemia cells [13]. Cotylenin A and fusicoccin A also act synergistically with interferon-α or rapamycin to induce apoptosis in cancer cell lines [14–16]. However, cotylenin A cannot be produced by its natural source, *Cladosporium sp.* 501-7W, due to the loss of its ability to proliferate during preservation [17]. The important biological activities and complex structures of fusicoccane diterpenoids have inspired several total syntheses, which range between 15 and 29 steps [18–26]. Most of these synthetic approaches rely on similar strategies, i.e., coupling of the A ring and the C ring followed by the formation of the B ring. Additionally, the semisynthesis of analogues of **1** has been reported and led to the discovery of ISIR-050 (**4**), which shows higher activity than cotylenin A in cell growth inhibition assays and less toxicity in single-agent treatments [27–28]. Recently, Jiang and Renata described a chemoenzymatic approach that combines the skeletal construction by chemical methods and enzymatic C–H oxidations [29]. The synthesis employs a catalytic Nozaki–Hiyama–Kishi reaction and a one-pot Prins cyclization/transannular hydride transfer to construct the 5-8-5 tricyclic scaffold. Enzymatic oxidations were used to install the hydroxy group at the C-3 position. Ten fusicoccanes were synthesized in 8–13 steps each. Despite these efforts, a strategy with limited chemical transformations is highly desirable and should enable the discovery of new fusicoccane derivatives with improved biological activity.

Inspired by the biosynthetic machinery of terpenoids, we have reported a hybrid synthetic strategy for accessing bioactive terpenoids by combining enzymatic terpene cyclization and chemical synthesis [30–33]. Briefly, the carbon scaffolds are forged by terpene cyclases, followed by concise chemical transformations to yield the desired natural products. Here, we describe heterologous biosynthesis of cotylenol by engineering the biosynthetic pathway of brassicicenes in *Aspergillus oryzae* and harnessing the promiscuity of a cytochrome P450 from the biosynthesis of fusicoccin A (Figure 1b). A key intermediate, brassicicenes I (**5**), was further used to achieve the collective synthesis of alterbrassicicene E (**6**), brassicicenes A (**7**) and R (**8**).

## Results and Discussion

**Figure 2:**
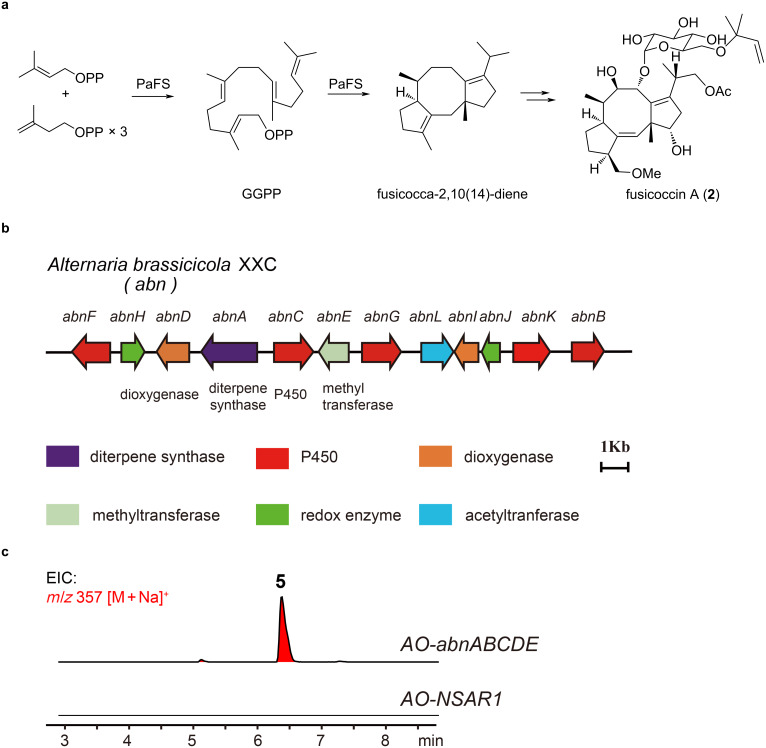
Heterologous production of brassicicene I in an engineered *A. oryzae* strain. (a) Biosynthesis of fusicoccin A in *Phomopsis (Fusicoccum) amygdali*. (b) Brassicicene BGC in *A. brassicicola* XXC. (c) Heterologous production of brassicicene I (**5**) in an engineered AO strain.

Fusicoccanes feature a characteristic dicyclopenta[*a*,*d*]cyclooctane (5-8-5) ring system that is biosynthesized from geranylgeranyl pyrophosphate (GGPP) via class I terpene cyclization (Figure 2a). To date, two fusicoccadiene synthases have been identified by the analysis of the brassicicene biosynthesis-related gene cluster (BGC) in *Alternaria brassicicola* and *Pseudocercospora fijiensis* [34–35]. The 5-8-5 tricyclic scaffold is transformed into various fusicoccane natural products catalyzed by P450s, dioxygenases, dehydrogenases, and reductases. Therefore, we propose to harness the biosynthetic pathway for brassicicenes, which share the same carbon skeleton and similar oxidation and unsaturation states as cotylenol and cotylenin A [36]. In a previous study, Oikawa and co-workers reported the identification of brassicicene BGC in *Pseudocercospora fijiensis* [37]. By heterologous expression of this BGC in *Aspergillus oryzae*, brassicicene I was produced by the transformant *AO-bscABCDE* at a titer of 5.5 mg/L. Recently, we identified a new BGC for brassicicenes, namely, *abn*, from the brassicicene-producing strain *A. brassicicola* XXC (Figure 2b) [38]. We constructed an *A. oryzae* strain with the homologous gene *abnABCDE*. As expected, compound **5** was produced at a titer of 8 mg/L (Figure 2c). By co-fermenting with Amberlite XAD-16, an enhanced yield of 30 mg/L was achieved, thus allowing further transformation into other natural products.

**Figure 3:**
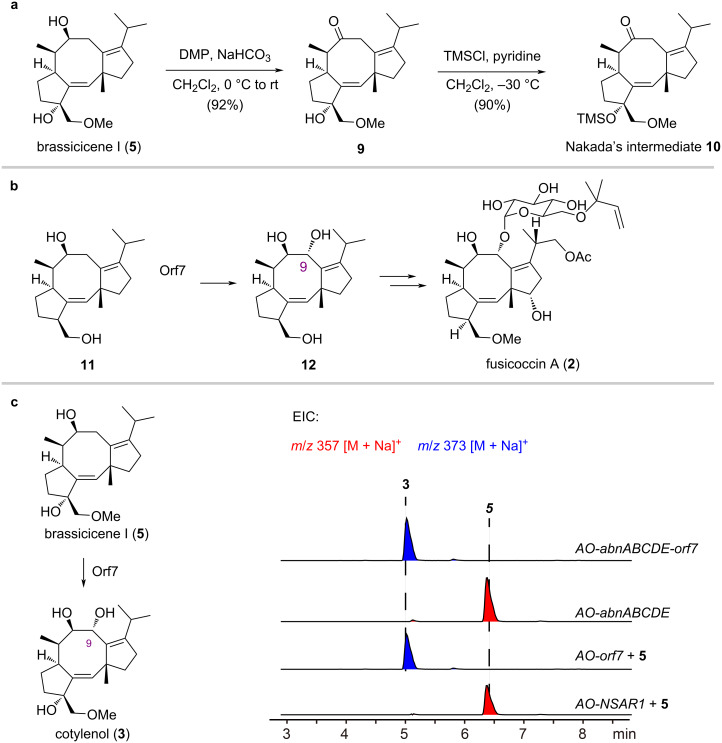
Synthesis of cotylenol (**3**). (a) Synthesis of Nakada’s intermediate **10** from **5**. (b) Orf7 catalyzes the oxidation of **11** in the biosynthesis of fusicoccin A (**2**). (c) LC–MS analysis of the production of **3** through *AO-abnABCDE+orf7* heterologous expression or *AO-orf7* biotransformation.

We next carried out the formal synthesis of cotylenin A and cotylenol (Figure 3a). Oxidation of brassicicene I with Dess–Martin reagent afforded intermediate **9** in 92% yield. The tertiary hydroxy group of compound **9** was further protected with a TMS group to provide compound **10** in 90% yield, a key intermediate in the synthesis of cotylenol and cotylenin A by Nakada and co-workers [21]. However, installing the C9 hydroxy group requires the use of stoichiometric MoOPH [39], which raises toxicity and safety issues. Therefore, we sought an enzymatic method to selectively oxidize **5** at the C9 position. Dairi and co-workers reported that Orf7 oxidizes compound **11** at the C9 position in the biosynthesis of fusicoccin A (Figure 3b) [40]. Given the structural similarities between compound **5** and compound **11**, we hypothesized that Orf7 might also catalyze the hydroxylation of compound **5** at C9. Hence, we fed an *A. oryzae* strain that expressed the *orf7* gene with compound **5**. To our delight, compound **3** was obtained successfully (Figure 3c). To stably produce **3** by fermentation, we constructed an *A. oryzae* strain that integrates *abnABCDE* with *orf7*, achieving a yield of 60 mg/kg rice through rice fermentation.

**Scheme 1:**
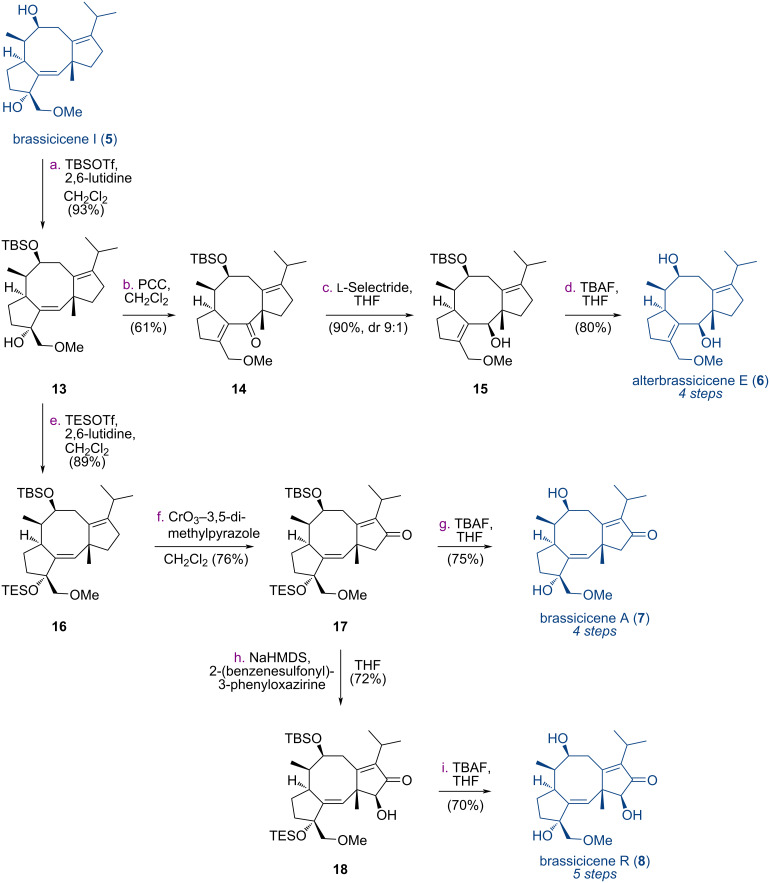
Synthesis of alterbrassicicene E (**6**) and brassicicenes A (**7**) and R (**8**) from brassicicene I (**5**).

We next targeted alterbrassicicene E (**6**), brassicicenes A (**7**) and R (**8**) (Scheme 1). The secondary hydroxy group of brassicicene I was selectively TBS-protected in the presence of TBSOTf and 2,6-lutidine to give compound **13** in 93% yield. Then, compound **13** underwent oxidative rearrangement with PCC to afford ketone **14** in 61% yield. Under Luche reduction conditions, compound **15** and its diastereomer were obtained in a total yield of 90% at a ratio of 1:0.7. To improve the diastereoselectivity, we examined other reduction conditions and found that ʟ-Selectride afforded compound **15** in 90% yield with a dr of 9:1. Upon desilylation with TBAF, compound **15** was converted into alterbrassicicene E (**6**) in 80% yield. To synthesize brassicicenes A (**7**) and R (**8**), the tertiary hydroxy group of compound **13** was protected with a TES group to furnish compound **16** in 89% yield. By screening several conditions, we found that allylic oxidation of compound **16** could be achieved in the presence of chromium trioxide–3,5-dimethylpyrazole complex [41] to provide compound **17** in 76% yield. After deprotection of the TBS and TES groups with TBAF, brassicicene A (**7**) was obtained in 75% yield. Compound **17** was subjected to α-hydroxylation from the less-hindered convex face using Davis’s oxaziridine [25], furnishing intermediate **18** in 72% yield. After deprotection of the TBS and TES groups, brassicicene R (**8**) was obtained in 70% yield. Therefore, alterbrassicicene E (**6**) and brassicicenes A (**7**) and R (**8**) were synthesized from brassicicene I over 4 or 5 chemical steps.

## Conclusion

In summary, the diverse biological activities and complex structures of fusicoccane diterpenoids have stimulated multiple elegant chemical syntheses. In contrast to these approaches, we harnessed the biosynthetic machinery of brassicicenes to produce brassicicene I in an engineered *A. oryzae* strain. Brassicicene I was further oxidized by a cytochrome P450 from the biosynthesis of fusicoccin A, thus leading to total biosynthesis of cotylenol in *A. oryzae*. Three fusicoccane diterpenoids, including alterbrassicicene E and brassicicenes A and R, were efficiently synthesized from brassicicene I in 4 or 5 chemical steps. This work lays the foundation for the preparation of fusicoccane natural products and exploration of their biological activities.

## Supporting Information

File 1 Experimental data and copies of spectra.

## Data Availability

All data that supports the findings of this study is available in the published article and/or the supporting information of this article.
