# Supplementary material for: Heterologous biosynthesis of cotylenol and concise synthesis of fusicoccane diterpenoids
Source: Beilstein J Org Chem. 2025 Jul 21;21:1489–95. doi: 10.3762/bjoc.21.111 (PMC12302409; doi:10.3762/bjoc.21.111)
Supplement: File 1 — Experimental data and copies of spectra. [file Beilstein_J_Org_Chem-21-1489-s001.pdf]

BEILSTEIN JOURNAL OF ORGANIC CHEMISTRY

# Supporting Information

for

# Heterologous biosynthesis of cotylenol and concise synthesis of fusicoccane diterpenoids

Ye Yuan, Zhenhua Guan, Xue-Jie Zhang, Nanyu Yao, Wenling Yuan, Yonghui Zhang, Ying Ye and Zheng Xiang

*Beilstein J. Org. Chem.* **2025,** *21,* 1489–1495. doi:10.3762/bjoc.21.111

# Experimental data and copies of spectra

License and Terms: This is a supporting information file under the terms of the Creative Commons Attribution License (https://creativecommons.org/licenses/by/4.0). Please note that the reuse, redistribution and reproduction in particular requires that the author(s) and source are credited and that individual graphics may be subject to special legal provisions.
The license is subject to the *Beilstein Journal of Organic Chemistry* terms and conditions: (https://www.beilstein-journals.org/bjoc/terms)

## Table of contents


**I. General information** S2

**II. Heterologous biosynthesis** S3

**III. Chemical synthesis** S9

**IV. References** S21

**V. NMR spectra** S22

## I. General information

HPLC–MS analyses were recorded on Agilent 1290 Infinity II-6545B Q-TOF. Oligonucleotides for PCRs were purchased from Sangon Biotech (Shanghai, China). Reagents were purchased from Bidepharm, Sigma Aldrich, Macklin, Merck and Aladdin and used without further purification. Anhydrous tetrahydrofuran and dichloromethane were purchased from Energy Chemical and used directly.

For reactions that require heating, an oil bath was used in all procedures. Reactions were monitored by thin-layer chromatography (TLC) carried out on 0.25 mm Tsingdao silica gel plates (GF-254) and visualized under UV light at 254 nm. Staining was performed with an ethanolic solution of phosphomolybdic acid (PMA) and subsequent heating. Tsingdao silica gel (200–300 mesh) was used for column chromatography. Yields refer to chromatographically and spectroscopically ($^1$H NMR) homogeneous materials.

NMR spectra were recorded on either a Bruker 400 MHz ($^1$H: 400 MHz, $^{13}$C: 100 MHz), Bruker 500 MHz ($^1$H: 500 MHz, $^{13}$C: 125 MHz) or Bruker 600 MHz ($^1$H: 600 MHz, $^{13}$C: 150 MHz). Chemical shifts are referenced to residual solvent ($CDCl_3$: $^1$H NMR = 7.26 ppm, $^{13}$C NMR = 77.16 ppm). The following abbreviations were used to explain the multiplicities: s = singlet, d = doublet, t = triplet, q = quartet, m = multiplet, br = broad. High-resolution mass spectra (HRMS) were measured on an ABI Q-star Elite with the ionization method ESI and the mass analyzer type of TOF. Optical rotation values were recorded on a Rudolph Research Analytical Autopol I polarimeter (Rudolph Research Co.).

Abbreviations: DCM: dichloromethane; EtOAc: ethyl acetate; PE: petroleum ether; DMSO: dimethyl sulfoxide; THF: tetrahydrofuran; equiv.: equivalent; TBAF: tetrabutylammonium fluoride trihydrate; PCC: pyridinium chlorochromate; M.P.: melting point; NMR: nuclear magnetic resonance.

## II. Heterologous biosynthesis of brassicicene I (5) and cotylenol (3)

**Strains**

*Escherichia coli* DH5α was used for cloning and following standard recombinant DNA techniques. *Aspergillus oryzae* NSAR1, a quadruple auxotrophic mutant (*niaD*$^{-}$, *sC*$^{-}$, *adeA*$^{-}$, and *argB*$^{-}$) was used as the host for gene expression. *Alternaria brassicicola* XXC was grown on potato dextrose agar at 30 °C for 4 days for gDNA extraction.

**Genomic DNA preparation**

Mycelia of *Alternaria brassicicola* XXC was grown in potato dextrose agar (PDA) at 30 °C for 4 days before it was collected and lyophilized with liquid nitrogen. The mycelia were grinded via Tissuelyser (Shanghai Jinxing Co., Ltd, China). Genomic DNA was extracted by fugal genomic DNA extraction kit (BioFlux) according to the instruction of the manufacturer.

**Construction of *A. oryzae* expression plasmids**

The primers used in this study are listed in Table S1. The *abnA, abnB, abnC, abnD, abnE* were amplified from the genomic DNA of *Alternaria brassicicola* XXC. The *orf7* gene was synthesized by GenScript (Nanjing, China). The linker region of the following plasmids was amplified from pAdeA2. Five expression plasmids were constructed as follows. The NheI digested fragments of pAdeA2 plasmid were subjected to Gibson assembly (NEBuilder HiFi DNA Assembly Master Mix, New England BioLabs) with *orf7* to construct pAdeA2-*orf7*. The KpnI and NheI digested fragments of pAdeA2 and pUARA2 plasmid were subjected to Gibson assembly (NEBuilder HiFi DNA Assembly Master Mix, New England BioLabs) with *abnA*, *abnB*, *abnC*, *abnD* and the corresponding linker to construct pAdeA2-*abnAB* and pUARA2-*abnCD*, respectively. The pUSA2-*abnE* and pUSA2-a*bnE-orf7* were also constructed by Gibson assembly.

**Transformation of *A. oryzae***

Transformation of *A. oryzae* was performed by the protoplast-polyethylene glycol method that has been reported.[1] Plasmids used for the construction of each transformant are summarized in Table S2.

**Extraction of the metabolites from the biotransformation *AO-abnABCDE and AO-abnABCDE-orf7***

Spore suspension of each transformant was inoculated into MPY medium (3% maltose, 1% hipolypeptone, 0.5% yeast extract, 0.925% $(NH_4)_2SO_4$, 100 mL) in 500 mL Erlenmeyer flasks. Each culture was incubated at 30 °C for 3 days. If appropriate, the mycelia and culture broth was separated prior to extraction. After the extraction with EtOAc, the extract was concentrated in vacuo to afford crude extracts. The crude extracts were analyzed by LC–MS with an Eclipse Plus C18 column (2.1 × 100 mm, 3.5 μm) under the following conditions. $CH_3CN$ and $H_2O$ (each contained 0.1% HCOOH) were used as eluents. The concentration of $CH_3CN$ was linearly increased from 5% to 98% over 11 min and kept at 100% for 2 min. Flow rate was kept at 0.5 mL min$^{-1}$. Metabolites were analyzed in ESI positive mode.

**Production of brassicicene I (5) from *AO-abnABCDE***

Spore suspension of transformant *AO-abnABCDE* was inoculated into MPY medium (3% maltose, 1% hipolypeptone, 0.5% yeast extract, 0.925% $(NH_4)_2SO_4$, 200 mL) in 1000 mL Erlenmeyer flasks. To each culture was added 3 g Amberlite XAD-16 packaged in tea bags. After incubating for 3 to 5 days at 30 °C, the Amberlite XAD-16 and liquid part were separated by filtration. The liquid part was extracted three times using EtOAc, and the Amberlite XAD-16 was soaked with MeOH for three times. The combined organic phase was concentrated in vacuo to afford crude extracts, which were further purified by column chromatography (silica gel, 200–300 mesh, PE/EtOAc 100:1 to 8:1) to afford brassicicene I (30 mg/L).

***Orf7* transformant feeding studies**

To test the gene function of *orf7 in vivo*, the *AO-NSAR1* and transformant *AO-orf7* were cultured on oat agar medium plates at 30 °C for 3 days, respectively, which then were grown in 4 mL MPY medium (3% maltose, 1% hipolypeptone, 0.5% yeast extract, 0.925% $(NH_4)_2SO_4$, 0.15% methionine, 0.06% arginine) with 100 μg brassicicene I (**5**). After incubating for 4 days at 30 °C, the mycelium and liquid part were separated by filtration. Then the liquid part was extracted three times using EtOAc. The solvent was removed under vacuo to obtain crude extracts, which were analyzed by LC–MS equipped with an Eclipse Plus C18 column (2.1 × 100 mm, 3.5 μm) under the

following conditions. $CH_3CN$ and $H_2O$ (each contained 0.1% HCOOH) were used as eluents. The concentration of $CH_3CN$ was linearly increased from 5% to 98% over 11 min and kept at 100% for 2 min. The flow rate was kept at 0.5 mL $min^{-1}$. Metabolites were analyzed in ESI positive mode.

**Production of cotylenol (3) from *AO-abnABCDE-orf7***

To produce cotylenol from *AO-abnABCDE-orf7*, the transformant *AO-abnABCDE-orf7* was cultured on oat agar medium plates at 30 °C for 3 days, which then was cultured with rice medium (1 kg) at 30 °C for 10 days and soaked with solvent EtOAc for three times. The solvent was removed in vacuo to obtain crude extracts, which were purified by column chromatography (silica gel, 200–300 mesh, PE/EtOAc 100:1 to 5:1) to afford cotylenol (**3**) (60 mg).

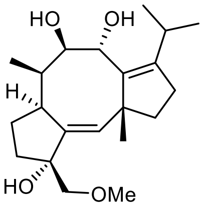


cotylenol (**3**)

Data for cotylenol (**3**):

white solid;

$[\alpha]_D^{25.0}$ = −88 (*c* 0.11, $CH_3OH$).

**$^1$H NMR** (400 MHz, $CDCl_3$) δ 5.51 (d, *J* = 2.6 Hz, 1H), 4.06 (d, *J* = 10.0 Hz, 1H), 3.93 (dd, *J* = 10.0, 4.4 Hz, 1H), 3.40 (s, 3H), 3.35 (d, *J* = 9.5 Hz, 1H), 3.26 (p, *J* = 6.8 Hz, 1H), 3.08 (dd, *J* = 9.5, 1.3 Hz, 1H), 2.93 (td, *J* = 8.6, 2.6 Hz, 1H), 2.17 – 2.06 (m, 3H), 2.03 – 1.92 (m, 3H), 1.84 (ddd, *J* = 12.0, 6.9, 2.2 Hz, 1H), 1.68 (ddd, *J* = 12.0, 10.0, 8.4 Hz, 1H), 1.41 (dddd, *J* = 13.3, 11.9, 7.7, 1.3 Hz, 1H), 1.33 – 1.27 (m, 1H), 1.21 (s, 3H), 1.03 (d, *J* = 6.7 Hz, 3H), 0.95 (d, *J* = 6.9 Hz, 3H), 0.80 (d, *J* = 7.2 Hz, 3H);

**$^{13}$C NMR** (150 MHz, $CDCl_3$) δ 150.4, 139.7, 136.9, 134.4, 82.1, 77.6, 77.4, 67.9, 59.4, 51.9, 42.6, 41.7, 40.3, 35.4, 31.7, 28.2, 27.2, 26.6, 21.6, 20.4, 8.5;

**HRMS (ESI)**: m/z calcd for $C_{21}H_{34}NaO_4^+$: 373.2349 $[M+Na]^+$; found: 373.2352.

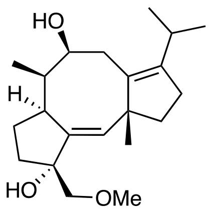

brassicicene I (**5**)

Data for brassicicene I (**5**):

colorless oil;

$[\alpha]_D^{25.0}$ = +27 (c 0.11, $CH_3OH$).

**$^1$H NMR** (600 MHz, $CDCl_3$) δ 5.59 (d, *J* = 2.5 Hz, 1H), 3.89 (dt, *J* = 11.5, 3.9 Hz, 1H), 3.41 (s, 3H), 3.38 (d, *J* = 9.5 Hz, 1H), 3.15 (d, *J* = 1.2 Hz, 1H), 2.89 (td, *J* = 8.0, 2.4 Hz, 1H), 2.78 (p, *J* = 6.8 Hz, 1H), 2.31 (dtd, *J* = 13.4, 2.5, 1.2 Hz, 1H), 2.22 – 1.92 (m, 5H), 1.80 (ddd, *J* = 12.0, 7.5, 3.3 Hz, 2H), 1.70 – 1.62 (m, 1H), 1.46 – 1.38 (m, 1H), 1.33 – 1.19 (m, 1H), 1.13 (s, 3H), 0.98 (dd, *J* = 12.5, 6.8 Hz, 6H), 0.80 (d, *J* = 7.1 Hz, 3H);

**$^{13}$C NMR** (150 MHz, $CDCl_3$) δ 146.0, 139.5, 135.6, 132.6, 82.1, 77.7, 76.4, 59.5, 52.9, 44.7, 42.1, 40.5, 35.7, 32.1, 28.6, 27.5, 27.2, 26.6, 21.0, 20.9, 8.1;

$^1$H and $^{13}$C NMR data are in good agreement with reported ones.[2]

**HRMS (ESI)**: m/z calcd for $C_{21}H_{34}NaO_3^+$: 357.2400 $[M+Na]^+$; found: 357.2397.

**Table S1:** Oligonucleotides used for construction of plasmids

| amplicon | Sequence (from 5' to 3') | size / vector |
|---|---|---|
| *abnA* | TTCGAATCGATTTGAGCTAGCATGAAATACCAATTTTCCATCA | 2524 bp |
| | CACTAGTGCGGCCGCTAGCTCAAAGCTTGAGCATCATTAG | pAdeA2 |
| *abnB* | CGGAATTCGAGCTCGGTACCATGGCTACAACCTTTACACA | 1972 bp |
| | ACTACAGATCCCCGGGTACCCTACTCCTTGTTTTTCTAACGA | pAdeA2 |
| linker | GGTACCCGGGGATCTGTAGT | 858 bp |
| | GCTAGCTCAAATCGATTCGA | |
| *abnC* | TCGAATCGATTTGAGCTAGCATGGCTTCCATACTATGGAC | 1760 bp |
| | GTCACTAGTGCGGCCGCTAGCTATTTCGTTCTCGGAGCGA | pUARA2 |
| *abnD* | CGGAATTCGAGCTCGGTACCATGGCAGTCCAAGAGACAGA | 1353 bp |
| | ACTACAGATCCCCGGGTACCTTATGCATTCTGTGCCGCAG | pUARA2 |
| *abnE* | CGGAATTCGAGCTCGGTACCATGGCTTCCACCAGTTCCAC | 1471 bp |
| | ACTACAGATCCCCGGGTACCTTAATGAGCCACCGCTGTTG | pUSA2 |
| *orf7* | AAGCTCCGAATTCGAATCGATTTGAGCTAGCATGCTCTCCACC ATGGAC | 1536 bp |
| | ACTACCCGGGTCACTAGTGCGGCCGCTAGCTCAACCTGGTAA CTTAACTTCCT | pUSA2 |

**Table S2:** Summary of the transformants in this study

| Transformants | plasmids | | |
|---|---|---|---|
| | *AdeA* | *ArgB* | *sC* |
| *AO-orf7* | pAdeA2-*orf7* | | |
| *AO-abnABCDE* | pAdeA2-*abnAB* | pUARA2-*abnCD* | pUSA2-*abnE* |
| *AO-abnABCDE-orf7* | pAdeA2-*abnAB* | pUARA2-*abnCD* | pUSA2-*abnE*-orf7 |

## III. Chemical synthesis and analysis

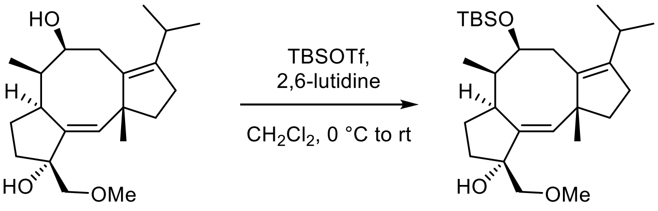


brassicicene I (**5**) **13**

To a stirred solution of brassicicene I (**5**, 237 mg, 0.71 mmol) and 2,6-lutidine (413 μL, 3.55 mmol) in $CH_2Cl_2$ (4 mL) was added *tert*-butyldimethylsilyl trifluoromethanesulfonate (TBSOTf, 490 μL, 2.13 mmol) at 0 °C. The reaction mixture was stirred for 2 hours until full consumption of brassicicene I. The resulting mixture was quenched with saturated $NH_4Cl$ solution (30 mL) and extracted with $CH_2Cl_2$ (50 mL × 3). The organic layers were combined and dried over $Na_2SO_4$ and filtered. The filtrate was concentrated under reduced pressure, and the residue was purified by column chromatography on silica gel (PE/EtOAc 30:1) to provide the desired product **13** (296 mg, 93%) as a light yellow oil.

Data for **13**:

$\mathbf{R_f}$ = 0.8 (PE : EtOAc = 3 : 1);

$[\alpha]_D^{20.0}$ = −12.00 (*c* 2.00, $CHCl_3$).

**$^1$H NMR** (400 MHz, $CDCl_3$) δ 5.57 (d, *J* = 2.4 Hz, 1H), 3.81 (dt, *J* = 11.2, 4.1 Hz, 1H), 3.41 (s, 3H), 3.39 (d, *J* = 9.7 Hz, 1H), 3.15 (d, *J* = 9.5 Hz, 1H), 2.86 (td, *J* = 7.9, 2.4 Hz, 1H), 2.76 (hept, *J* = 6.9 Hz, 1H), 2.41 (s, 1H), 2.25 – 2.13 (m, 1H), 2.12 – 2.01 (m, 3H), 2.00 – 1.89 (m, 2H), 1.77 (ddd, *J* = 11.4, 7.2, 3.6 Hz, 1H), 1.73 – 1.56 (m, 2H), 1.43 (dt, *J* = 13.9, 7.8 Hz, 1H), 1.32 – 1.19 (m, 1H), 1.10 (s, 3H), 0.99 (dd, *J* = 14.5, 6.8 Hz, 6H), 0.89 (s, 9H), 0.77 (d, *J* = 7.1 Hz, 3H), 0.09 (s, 3H), 0.06 (s, 3H) ppm.

**$^{13}$C NMR** (100 MHz, $CDCl_3$) δ 145.3, 139.8, 135.4, 133.7, 82.1, 77.8, 76.6, 59.5, 52.9, 45.6, 42.0, 40.5, 35.8, 32.5, 29.3, 27.7, 27.2, 26.6, 26.0, 20.9, 20.9, 18.2, 8.3, −4.4, −4.7 ppm.

**HRMS (ESI)**: m/z calcd for $C_{27}H_{48}NaO_3Si^+$: 471.3265 $[M+Na]^+$; found: 471.3268.

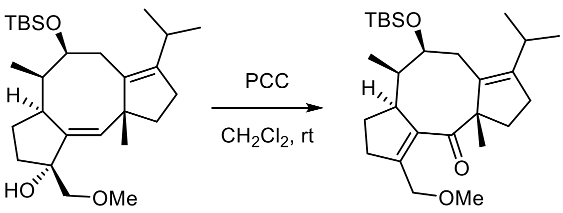

**13** **14**

To a solution of tertiary alcohols **13** (135 mg, 0.3 mmol) in $CH_2Cl_2$ (3.0 mL) was added PCC (97 mg, 0.45 mmol), and the mixture was stirred at room temperature for 12 hours. The reaction mixture was diluted with $CH_2Cl_2$ (60 mL) and filtered through a small silica gel column. Evaporation of the solvent and purification of the residue on a silica gel column (PE/EtOAc 50:1) furnished the enone **14** (82 mg, 61%) as a light yellow oil.

Data for **14**:

**R$_f$** = 0.6 (PE : EtOAc = 20 : 1);

**$[\alpha]_D^{20.0}$** = +33.32 (*c* 0.60, $CHCl_3$).

**$^1$H NMR** (400 MHz, $CDCl_3$) δ 4.05 – 3.91 (m, 2H), 3.87 (dt, *J* = 11.4, 4.1 Hz, 1H), 3.27 (s, 3H), 3.26 – 3.14 (m, 1H), 2.75 (p, *J* = 6.8 Hz, 1H), 2.40 (d, *J* = 9.3 Hz, 2H), 2.35 – 2.17 (m, 3H), 2.08 (dtd, *J* = 12.1, 7.8, 3.8 Hz, 1H), 1.99 (t, *J* = 12.7 Hz, 1H), 1.93 – 1.84 (m, 1H), 1.79 (p, *J* = 7.9, 7.1 Hz, 1H), 1.57 – 1.40 (m, 2H), 1.11 (d, *J* = 6.8 Hz, 3H), 1.08 (s, 3H), 0.98 (d, *J* = 6.8 Hz, 3H), 0.88 (s, 9H), 0.73 (d, *J* = 7.3 Hz, 3H), 0.08 (s, 3H), 0.05 (s, 3H) ppm.

**$^{13}$C NMR** (100 MHz, $CDCl_3$) δ 210.4, 145.6 (overlapping), 142.9, 134.8, 76.1, 69.7, 63.8, 58.6, 49.7, 34.9, 32.7, 31.9, 29.7, 28.3, 27.7, 25.9, 21.3, 20.9, 19.9, 18.1, −4.4, −4.7 ppm.

**HRMS (ESI)**: m/z calcd for $C_{27}H_{46}NaO_3Si^+$: 469.3108 [M+Na]$^+$; found: 469.3109.

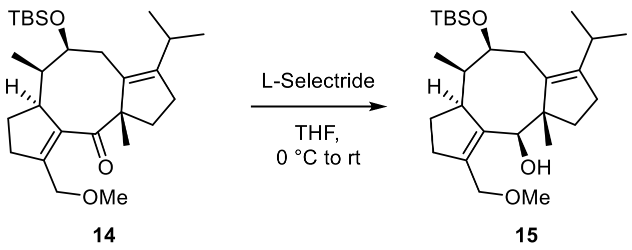

Enone **14** (41 mg, 0.09 mmol) in THF (3 mL) was cooled to 0 °C under argon. L-Selectride (1 M in THF, 0.27 mL, 0.27 mmol) was added dropwise. After the addition, the reaction mixture was allowed to warm to room temperature. After stirring for 1 hour, a hydrolytic work-up was carried out by using a saturated aqueous solution of $NH_4Cl$. The organic phase was extracted with ethyl acetate. The combined organic extracts were dried over $Na_2SO_4$, filtered and the solvent was removed under reduced pressure. The crude product showed a 9:1 d.r. as determined by $^1H$ NMR spectroscopy. The yield of diastereoisomers is 90% after flash column chromatography (PE/EtOAc 8:1). The crude product was purified by silica gel chromatography (PE/EtOAc 25:1) to give the desired product **15** (31 mg, 75%) as a colorless oil.

Data for **15**:

**$R_f$** = 0.55 (PE : EtOAc = 5 : 1);

**$[\alpha]_D^{20.0}$** = −22.21 (*c* 3.60, $CHCl_3$).

**$^1H$ NMR** (400 MHz, $CDCl_3$) δ 4.40 (dd, *J* = 11.5, 2.1 Hz, 1H), 4.05 (d, *J* = 3.6 Hz, 1H), 3.95 (d, *J* = 1.7 Hz, 1H), 3.72 (ddd, *J* = 11.4, 5.8, 3.0 Hz, 1H), 3.36 (s, 3H), 3.24 (d, *J* = 4.0 Hz, 1H), 2.70 (s, 1H), 2.56 (p, *J* = 6.8 Hz, 1H), 2.33 – 2.19 (m, 6H), 2.10 (dd, *J* = 13.2, 11.5 Hz, 1H), 1.97 – 1.81 (m, 4H), 1.08 – 0.55 (m, 12H), 0.89 (s, 9H), 0.06 (s, 3H), 0.04 (s, 3H) ppm.

**$^{13}C$ NMR** (100 MHz, $CDCl_3$) δ 144.8, 144.7, 135.1, 134.2, 78.5, 71.2, 58.7, 57.0, 55.7, 36.5, 35.2, 33.9, 32.2, 29.8, 27.9, 27.1, 26.1, 24.8, 21.8, 20.9, 19.4, 19.0, 18.1, −3.9, −4.9 ppm.

**HRMS (ESI)**: m/z calcd for $C_{27}H_{48}NaO_3Si^+$: 471.3265 [M+Na]$^+$; found: 471.3269.

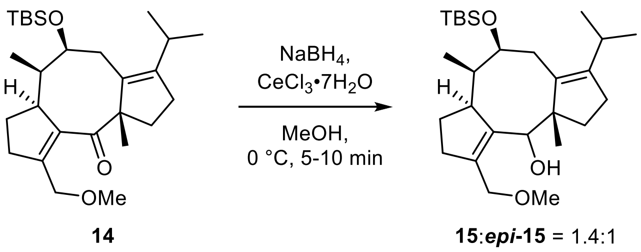

The starting enone **14** (41 mg, 0.09 mmol) was dissolved in 1 mL methanol at 0 °C. Then, $CeCl_3 \cdot 7H_2O$ (22.4 mg, 0.06 mM) and $NaBH_4$ (7.0 mg, 0.18 mmol) was slowly added with stirring. The mixture was allowed to react for 5–10 min until full consumption of enone **14**, then treated with water and extracted with EtOAc. The organic layers were combined and dried over $Na_2SO_4$ and filtered. The filtrate was concentrated under reduced pressure, and the residue was purified by column chromatography on silica gel (PE/EtOAc 20:1) to give the corresponding alcohol (37 mg, 91%) in 1.4:1 d.r.

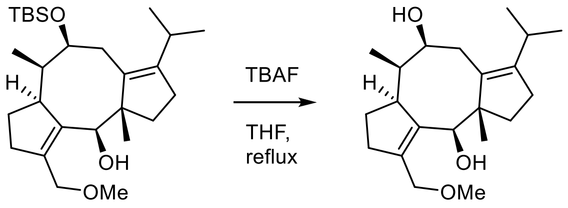


**15** alterbrassicicene E (**6**)

To a stirred solution of **15** (16.0 mg, 0.036 mmol) in THF (1.0 mL) in a Schlenk tube under argon was added tetrabutylammonium fluoride (TBAF, 1.0 mol/L in THF, 0.11 mmol, 110 μL) at room temperature. The mixture was stirred at 70 °C for 12 hours until full conversion of **15**. The mixture was quenched with a saturated aqueous solution of $NH_4Cl$ (5 mL) and extracted with EtOAc (3 × 25 mL). The combined organic layers were washed with brine, dried over anhydrous $Na_2SO_4$, filtered, and concentrated under reduced pressure. The residue was purified by column chromatography (PE/EtOAc 4:1) to provide alterbrassicicene E (**6**) (9.5 mg, 80%) as a white solid.

Data for alterbrassicicene E (**6**):

$\mathbf{R_f}$ = 0.55 (PE : EtOAc = 2 : 1);

$[\alpha]_D^{20.0}$ = +6.66 (*c* 0.60, $CHCl_3$).

$^{1}$**H NMR** (400 MHz, $CD_3OD$) δ 4.43 – 4.15 (m, 2H), 4.02 (s, 1H), 3.86 – 3.61 (m, 1H), 3.31 (s, 3H), 2.82 – 2.59 (m, 2H), 2.47 – 2.33 (m, 2H), 2.21 (dt, *J* = 19.0, 7.9 Hz, 5H), 2.03 – 1.70 (m, 4H), 1.07 (d, *J* = 7.3 Hz, 3H), 1.05 – 0.98 (m, 6H), 0.95 (d, *J* = 6.8 Hz, 3H) ppm.

$^{13}$**C NMR** (100 MHz, $CD_3OD$) δ 146.0, 144.7, 137.4, 135.5, 79.1, 77.1, 71.8, 58.4, 57.2, 56.4, 37.7, 34.8, 34.2, 32.3, 28.6, 27.7, 25.8, 22.0, 21.1, 19.2, 18.9 ppm.

**HRMS (ESI)**: m/z calcd for $C_{27}H_{48}NaO_3Si^+$: 357.2400 $[M+Na]^+$; found: 357.2402.

The spectra data was consistent with the literature.[3]

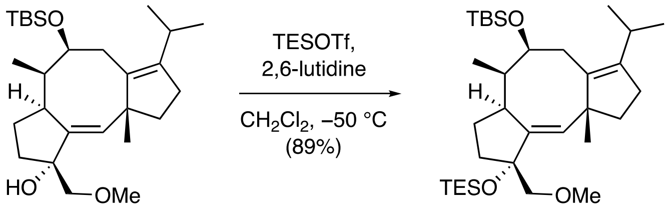

13 16

To a stirred solution of **13** (135 mg, 0.3 mmol) and 2,6-lutidine (105 μL, 0.9 mmol) in $CH_2Cl_2$ (4 mL) at −50 °C was added triethylsilyl trifluoromethanesulfonate (TESOTf; 132 μL, 0.6 mmol). The reaction mixture was stirred at −50 °C for 2 h. The reaction was quenched with saturated $NH_4Cl$ (40 mL) solution and extracted with $CH_2Cl_2$ (50 mL × 3). The organic layers were combined and dried over $Na_2SO_4$ and filtered. The filtrate was concentrated under reduced pressure, and the residue was purified by column chromatography on silica gel (PE/EtOAc 100:1) to give protected alcohol **16** (150 mg, 89%) as colorless oil.

Data for **16**:

**$R_f$** = 0.8 (PE : EtOAc = 25 : 1);

**$[\alpha]_D^{20.0}$** = −16.24 (*c* 3.20, $CHCl_3$).

**$^1$H NMR** (400 MHz, $CDCl_3$) δ 5.46 (d, *J* = 2.6 Hz, 1H), 3.79 (dt, *J* = 11.2, 4.1 Hz, 1H), 3.35 (s, 3H), 3.31 (d, *J* = 10.4 Hz, 1H), 2.91 (dd, *J* = 10.4, 1.5 Hz, 1H), 2.82 – 2.63 (m, 2H), 2.37 – 1.92 (m, 5H), 1.93 – 1.75 (m, 2H), 1.65 – 1.53 (m, 2H), 1.46 – 1.30 (m, 1H), 1.23 – 1.12 (m, 1H), 1.12 (s, 3H), 1.02 – 0.90 (m, 15H), 0.90 (s, 9H), 0.77 (d, *J* = 7.1 Hz, 3H), 0.57 (h, *J* = 7.6 Hz, 6H), 0.09 (s, 3H), 0.06 (s, 3H) ppm.

**$^{13}$C NMR** (100 MHz, $CDCl_3$) δ 145.5, 139.8, 134.6, 133.2, 85.0, 77.7, 76.8, 59.4, 52.7, 45.0, 42.7, 40.2, 34.1, 31.8, 29.3, 27.7, 27.3, 26.4, 26.0, 21.0, 21.0, 18.2, 7.7, 7.4, 6.8, −4.4, −4.7 ppm.

**HRMS (ESI)**: m/z calcd for $C_{33}H_{62}NaO_3Si_2^+$: 585.4130 [M+Na]$^+$; found: 585.4132.

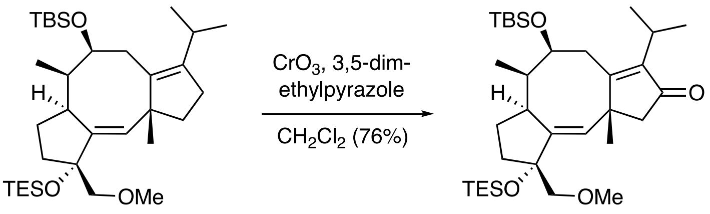

**16** **17**

3,5-Dimethylpyrazole (327 mg, 3.4 mmol) was added to a suspension of $CrO_3$ (340 mg, 3.4 mmol) in $CH_2Cl_2$ (15 mL) at 0 °C. After being stirred at 0 °C for 15 min, this mixture was added to a solution of **16** (160 mg, 0.28 mmol) in $CH_2Cl_2$ (15 mL) at room temperature. The reaction mixture was stirred at room temperature for 4 to 6 hours. Prolonging the reaction time will increase by-products. After the complete consumption of starting material **16**, silica gel (4 g) was added. The resultant suspension was concentrated, charged on silica gel and purified (PE/EtOAc 80:1) to afford **17** (123 mg, 76%) as a light yellow oil.

Data for **17**:

**R*f*** = 0.4 (PE : EtOAc = 20 : 1);

$[\alpha]_D^{20.0}$ = −3.08 (*c* 0.80, $CHCl_3$).

**$^1$H NMR** (400 MHz, $CDCl_3$) δ 5.64 (d, *J* = 2.5 Hz, 1H), 3.88 (dt, *J* = 11.2, 3.6 Hz, 1H), 3.35 (d, *J* = 10.0 Hz, 1H), 3.33 (s, 3H), 3.00 (dd, *J* = 10.1, 1.2 Hz, 1H), 2.74 (p, *J* = 7.0 Hz, 1H), 2.63 – 2.50 (m, 2H), 2.42 (dd, *J* = 12.9, 3.1 Hz, 1H), 2.36 (d, *J* = 18.2 Hz, 1H), 2.24 (dd, *J* = 18.2, 0.9 Hz, 1H), 2.02 (ddd, *J* = 11.9, 6.3, 3.2 Hz, 1H), 1.88 (dddd, *J* = 12.7, 9.1, 7.1, 3.2 Hz, 1H), 1.75 – 1.69 (m, 1H), 1.39 – 1.22 (m, 6H), 1.19 (d, *J* = 6.9 Hz, 3H), 1.00 – 0.77 (m, 21H), 0.54 (qd, *J* = 7.9, 5.3 Hz, 6H), 0.11 (s, 3H), 0.07 (s, 3H) ppm.

**$^{13}$C NMR** (100 MHz, $CDCl_3$) δ 207.0, 172.6, 146.0, 140.4, 133.2, 84.7, 77.2, 76.3, 59.1, 53.1, 47.2, 45.3, 39.9, 34.6, 31.5, 31.4, 27.6, 26.6, 25.9, 19.9, 19.7, 18.1, 7.5, 7.4, 6.7, −4.5, −4.7 ppm.

**HRMS (ESI)**: m/z calcd for $C_{33}H_{60}NaO_4Si_2^+$: 599.3922 [M+Na]$^+$; found: 599.3924.

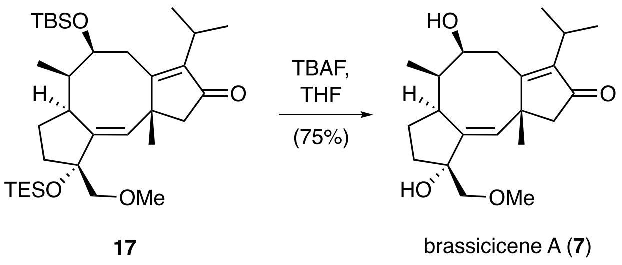

To a stirred solution of **17** (14.0 mg, 0.024 mmol) in THF (0.8 mL) in a Schlenk tube under argon was added tetrabutylammonium fluoride (TBAF, 1.0 mol/L in THF, 0.072 mmol, 72 μL) at room temperature. The mixture was stirred at 50 °C for 1 hour until the fully conversion of **17**. The mixture was quenched with a saturated aqueous solution of $NH_4Cl$ (5 mL) and extracted with EtOAc (3 × 25 mL). The combined organic layers were washed with brine, dried over anhydrous $Na_2SO_4$, filtered, and concentrated under reduced pressure. The residue was purified by column chromatography (PE/EtOAc 1:1) to provide brassicicene A (**7**) (6.3 mg, 75%) as a white solid.

Data for brassicicene A (**7**):

**$R_f$** = 0.16 (PE : EtOAc = 1 : 1);

**$[\alpha]_D^{20.0}$** = +43.32 (*c* 3.60, $CHCl_3$).

**$^1$H NMR** (400 MHz, $CD_3OD$) δ 5.75 (d, *J* = 2.3 Hz, 1H), 3.94 (dt, *J* = 10.3, 4.0 Hz, 1H), 3.44 (d, *J* = 10.0 Hz, 1H), 3.39 (s, 3H), 3.28 (d, *J* = 9.9 Hz, 1H), 2.86 (p, *J* = 6.9 Hz, 1H), 2.71 (ddd, *J* = 8.5, 5.9, 2.2 Hz, 1H), 2.64 – 2.53 (m, 2H), 2.47 (d, *J* = 18.6 Hz, 1H), 2.28 (d, *J* = 18.8 Hz, 1H), 2.03 – 1.82 (m, 3H), 1.57 – 1.43 (m, 1H), 1.42 – 1.36 (m, 1H), 1.33 (s, 3H), 1.26 (d, J = 7.0 Hz, 3H), 1.17 (d, J = 6.9 Hz, 3H), 0.91 (d, J = 7.0 Hz, 3H) ppm.

**$^{13}$C NMR** (100 MHz, $CD_3OD$) δ 209.7, 175.9, 146.9, 142.1, 134.7, 83.3, 78.2, 76.4, 59.6, 53.4, 48.5, 46.1, 42.2, 36.5, 32.8, 31.4, 28.1, 27.3, 20.0, 19.9, 8.4 ppm.

**HRMS (ESI)**: m/z calcd for $C_{21}H_{32}NaO_4^+$: 371.2193 $[M+Na]^+$; found: 371.2191.

The spectra data was consistent with the literature report.[4]

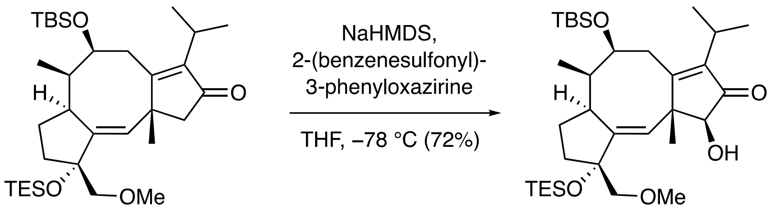


**17** **18**

To a stirred solution of **17** (40.0 mg, 0.07 mmol) in THF (2 mL) under argon was added NaHMDS (1 M in THF, 0.35 mL, 0.35 mmol) at −78 °C. The mixture was stirred at the same temperature for 0.5 h followed by the addition of 2-(benzenesulfonyl)-3-phenyloxaziridine (55 mg, 0.21 mmol). The mixture was stirred for 4 hours at −78 °C before it was quenched with a saturated aqueous solution of $Na_2S_2O_4$ (15 mL). The resulting mixture was extracted with EtOAc (3 × 50 mL). The combined organic layers were washed with brine (2.0 mL), dried over anhydrous $Na_2SO_4$, filtered, and concentrated under reduced pressure. The residue was purified by column chromatography (PE/EtOAc 30:1) to provide product **18** (30 mg, 72%) as a light yellow oil.

Data for **18**:

**R*f*** = 0.4 (PE : EtOAc = 12.5 : 1);

$[\alpha]_D^{20.0}$ = +4.44 (*c* 1.80, $CHCl_3$).

**$^1$H NMR** (400 MHz, $CDCl_3$) δ 5.82 (d, *J* = 2.2 Hz, 1H), 3.87 – 3.79 (m, 2H), 3.43 (d, *J* = 10.0 Hz, 1H), 3.35 (s, 3H), 3.16 (d, *J* = 9.9 Hz, 1H), 2.76 (p, *J* = 7.0 Hz, 1H), 2.64 – 2.47 (m, 3H), 2.41 (dd, *J* = 12.7, 2.5 Hz, 1H), 2.11 – 1.82 (m, 2H), 1.79 – 1.69 (m, 1H), 1.51 – 1.32 (m, 2H), 1.26 (d, *J* = 7.0 Hz, 3H), 1.20 (d, *J* = 7.0 Hz, 3H), 1.13 (s, 3H), 0.99 – 0.84 (m, 21H), 0.56 (qd, *J* = 7.9, 5.2 Hz, 6H), 0.10 (s, 3H), 0.07 (s, 3H) ppm.

**$^{13}$C NMR** (100 MHz, $CDCl_3$) δ 207.1, 173.3, 143.6, 141.8, 131.7, 84.8, 82.4, 77.6, 59.1, 51.9, 44.3, 40.3, 35.5, 32.3, 31.6, 26.2, 25.9, 23.7, 20.1, 19.5, 18.1, 7.3, 6.7, −4.6, −4.6 ppm.

**HRMS (ESI)**: m/z calcd for $C_{33}H_{60}NaO_5Si_2^+$: 615.3871 $[M+Na]^+$; found: 615.3873.

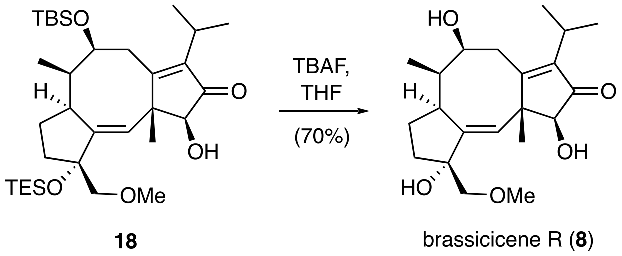

To a stirred solution of **18** (15.0 mg, 0.025 mmol) in THF (0.8 mL) in a Schlenk tube under argon was added tetrabutylammonium fluoride (TBAF, 1.0 mol/L in THF, 0.25 mmol, 250 μL) at room temperature. The mixture was stirred at room temperature for 2 hours until full conversion of **18**. The mixture was quenched with a saturated aqueous solution of $NH_4Cl$ (5 mL) and extracted with EtOAc (3 × 20 mL). The combined organic layers were washed with brine, dried over anhydrous $Na_2SO_4$, filtered, and concentrated under reduced pressure. The residue was purified by column chromatography (100% EtOAc) to provide brassicicene R (**8**) (6.4 mg, 70%) as a white solid.

Data for brassicicene R (**8**):

**R*f*** = 0.5 (EtOAc);

$[\alpha]_D^{20.0}$ = +40.00 (*c* 0.60, $CHCl_3$).

**$^1$H NMR** (400 MHz, $CD_3OD$) δ 5.84 (d, *J* = 2.1 Hz, 1H), 3.91 (s, 1H), 3.87 (dt, *J* = 9.1, 4.2 Hz, 1H), 3.51 (d, *J* = 9.8 Hz, 1H), 3.45 – 3.37 (m, 4H), 2.88 (p, *J* = 6.9 Hz, 1H), 2.64 (dd, *J* = 7.7, 4.3 Hz, 1H), 2.57 – 2.45 (m, 2H), 2.21 – 1.98 (m, 2H), 1.97 – 1.87 (m, 1H), 1.68 – 1.54 (m, 1H), 1.54 – 1.44 (m, 1H), 1.24 (d, *J* = 6.9 Hz, 3H), 1.19 (d, *J* = 7.0 Hz, 3H), 1.14 (s, 3H), 0.93 (d, *J* = 7.0 Hz, 3H) ppm.

**$^{13}$C NMR** (100 MHz, $CD_3OD$) δ 208.1, 173.8, 145.3, 143.5, 133.6, 83.8, 83.3, 78.7, 77.3, 59.7, 53.1, 45.5, 42.5, 37.3, 33.4, 31.9, 26.9, 24.2, 20.4, 19.5, 8.9 ppm.

**HRMS (ESI)**: m/z calcd for $C_{21}H_{32}NaO_5^+$: 387.2142 $[M+Na]^+$; found: 387.2142.

The spectra data was consistent with the literature report.[4]

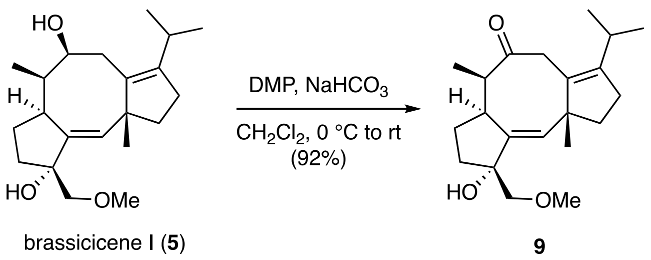


To a stirred solution of brassicicene I (**5**, 20 mg, 0.06 mmol) in 2.0 mL $CH_2Cl_2$ at 0 °C was added $NaHCO_3$ (30 mg, 0.36 mmol) and Dess–Martin periodinane (51 mg, 0.12 mmol). The solution was stirred at 0 °C for 15 minutes and at room temperature for 1 hour. After the complete conversion of brassicicene I (**5**), the reaction was quenched with 30 mL of a 1:1 mixture of 1 M $Na_2S_2O_3$ and saturated $NaHCO_3$ solution. The mixture was stirred until both layers were clear. The aqueous layer was extracted with $CH_2Cl_2$ (3 × 30 mL). The organic layers were combined, dried with $Na_2SO_4$, filtered and the solvent was evaporated under reduced pressure. The crude product was purified by flash column chromatography (PE/EtOAc 10:1) to give the ketone **9** (18 mg, 92%) as a light yellow oil.

Data for **9**:

**$R_f$** = 0.45 (PE : EtOAc = 3 : 1);

**$[\alpha]_D^{20.0}$** = +41.16 (*c* 3.40, $CHCl_3$).

**$^1$H NMR** (400 MHz, $CDCl_3$) δ 5.62 (d, *J* = 2.4 Hz, 1H), 3.67 (dddd, *J* = 8.1, 6.2, 3.8, 2.3 Hz, 1H), 3.39 (s, 3H), 3.33 (d, *J* = 14.6 Hz, 1H), 3.29 (d, *J* = 9.5 Hz, 1H), 3.21 (d, *J* = 9.5 Hz, 1H), 2.97 (p, *J* = 6.9 Hz, 1H), 2.90 (dt, *J* = 14.6, 2.4 Hz, 1H), 2.76 (p, *J* = 6.8 Hz, 1H), 2.42 (s, 1H), 2.22 (ddd, *J* = 9.7, 6.0, 2.6 Hz, 2H), 2.02 – 1.87 (m, 2H), 1.85 – 1.70 (m, 2H), 1.62 – 1.47 (m, 2H), 1.12 (s, 3H), 1.07 – 0.86 (m, 9H) ppm.

**$^{13}$C NMR** (100 MHz, $CDCl_3$) δ 212.3, 145.1, 143.2, 135.5, 132.7, 81.8, 78.7, 59.5, 53.3, 49.1, 41.1, 40.4, 40.3, 36.7, 27.8, 27.7, 27.5, 27.2, 21.2, 20.3, 12.2 ppm.

**HRMS (ESI)**: m/z calcd for $C_{21}H_{32}NaO_3^+$: 355.2244 $[M+Na]^+$; found: 355.2246.

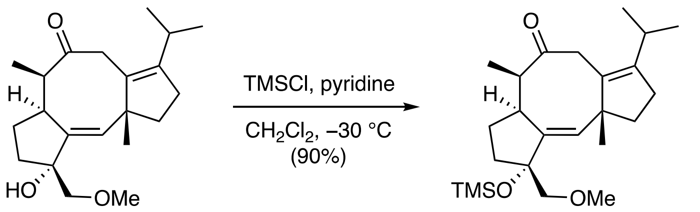


**9** Nakada's intermediate (**10**)

To a stirred solution of **9** (20 mg, 0.06 mmol) and pyridine (15 µL, 0.18 mmol) in $CH_2Cl_2$ (2 mL) at −30 °C was added chlorotrimethylsilane (TMSCl, 15 µL, 0.12 mmol). The reaction mixture was stirred at −30 °C for 15 minutes. The reaction was quenched with saturated $NH_4Cl$ (40 mL) solution and extracted with $CH_2Cl_2$ (50 mL × 3). The organic layers were combined and dried over $Na_2SO_4$ and filtered. The filtrate was concentrated under reduced pressure, and the residue was purified by column chromatography on silica gel (PE/EtOAc 20:1) to give Nakada's intermediate **10** (22 mg, 90%) as a yellow oil.

Data for **10**:

**$R_f$** = 0.6 (PE : EtOAc = 8 : 1);

**$[\alpha]_D^{20.0}$** = −25.71 (*c* 1.40, $CHCl_3$).

**$^1$H NMR** (400 MHz, $CDCl_3$) δ 5.51 (d, *J* = 2.3 Hz, 1H), 3.35 (s, 3H), 3.31 – 3.23 (m, 2H), 3.20 (d, *J* = 15.6 Hz, 1H), 3.11 (d, *J* = 10.5 Hz, 1H), 2.99 (dd, *J* = 10.5, 1.1 Hz, 1H), 2.81 (dt, *J* = 15.7, 2.0 Hz, 1H), 2.72 (p, *J* = 6.9 Hz, 1H), 2.25 (t, *J* = 6.7 Hz, 2H), 2.14 – 2.04 (m, 1H), 1.92 – 1.69 (m, 3H), 1.52 – 1.33 (m, 2H), 1.11 (s, 3H), 1.07 – 0.88 (m, 9H), 0.08 (s, 9H) ppm.

**$^{13}$C NMR** (100 MHz, $CDCl_3$) δ 213.7, 145.1, 143.6, 134.1, 134.0, 85.2, 78.3, 59.4, 52.3, 45.3, 40.9, 40.6, 39.9, 34.3, 27.8, 27.5, 27.4, 26.6, 21.2, 20.3, 12.7, 2.5 ppm.

**HRMS (ESI)**: m/z calcd for $C_{24}H_{40}NaO_3Si^+$: 427.2639 $[M+Na]^+$; found: 427.2637.

The spectra data was consistent with the literature report.[5]

## IV. References


1. Liu, C.; Tagami, K.; Minami, A.; Matsumoto, T.; Frisvad, J. C.; Suzuki, H.; Ishikawa, J.; Gomi, K.; Oikawa, H. Reconstitution of Biosynthetic Machinery for the Synthesis of the Highly Elaborated Indole Diterpene Penitrem. *Angew. Chem. Int. Ed.* **2015**, *54*, 5748–5752. DOI: 10.1002/anie.201501072.
2. Pedras, M. S.; Chumala, P. B.; Jin, W.; Islam, M. S.; Hauck, D. W. The phytopathogenic fungus Alternaria brassicicola: Phytotoxin production and phytoalexin elicitation. *Phytochemistry*. **2009**, *70*, 394–402. DOI: 10.1016/j.phytochem.2009.01.005
3. Li, F.; Lin, S.; Zhang, S.; Pan, L.; Chai, C.; Su, J.; Yang, B.; Liu, J.; Wang, J.; Hu, Z.; Zhang, Y. Modified Fusicoccane-Type Diterpenoids from *Alternaria brassicicola*. *J. Nat. Prod.* **2020**, *83*, 1931–1938. DOI: 10.1021/acs.jnatprod.0c00165
4. Chen, B.; Yang, Y.; Zhang, X.; Xu, D.; Sun, Y.; Chen, Y.; Wang, L. Total Syntheses of Brassicicenes A, R, and T. *Org. Lett.* **2023**, *25*, 8570–8574. DOI: 10.1021/acs.orglett.3c033555.
5. Uwamori, M.; Osada, R.; Sugiyama, R.; Nagatani, K.; Nakada, M. Enantioselective Total Synthesis of Cotylenin A. *J. Am. Chem. Soc.* **2020**, *142*, 5556–5561. DOI: 10.1021/jacs.0c01774.

## V. NMR Spectra

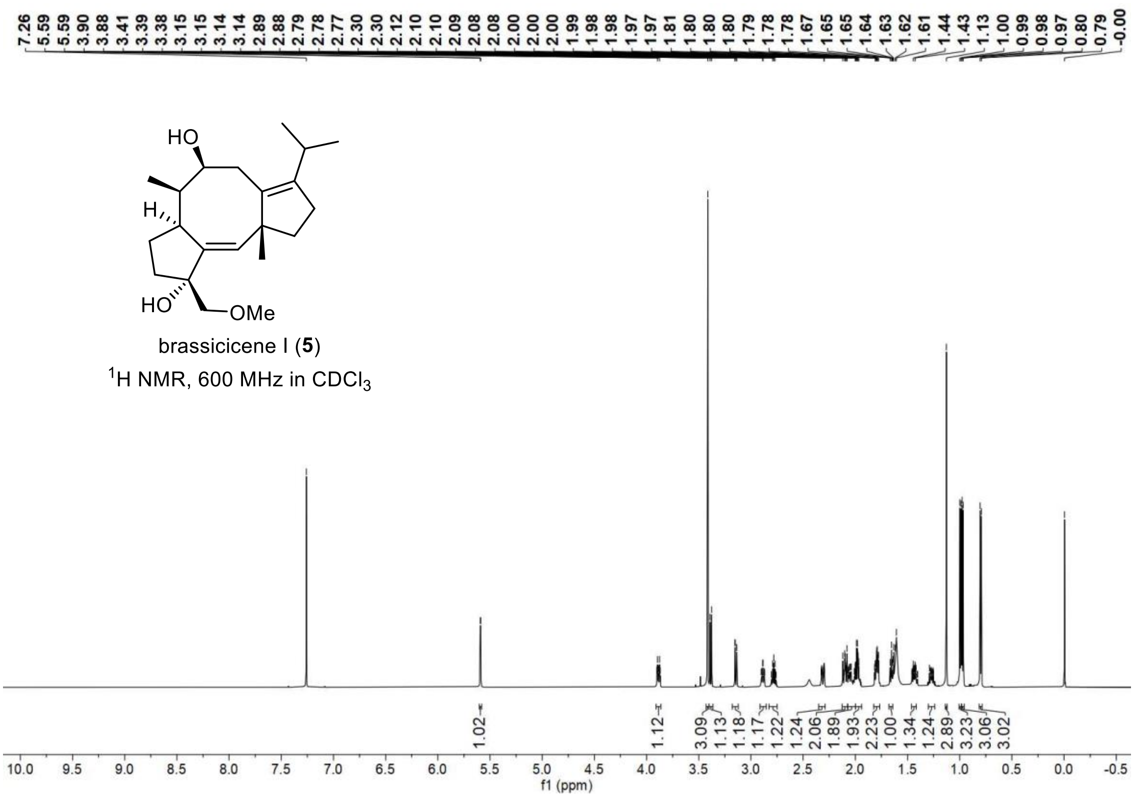


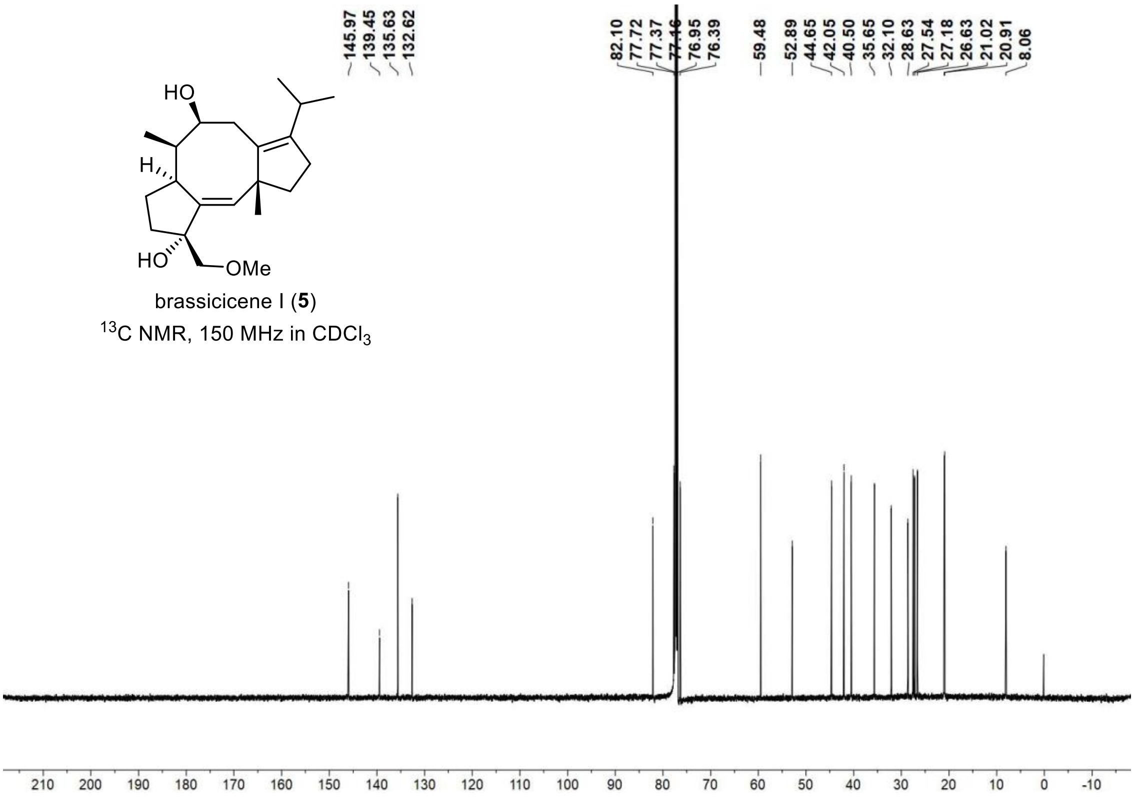

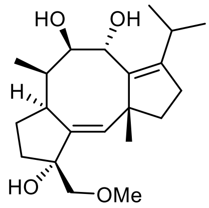


Cotylenol (**3**)
$^{1}$H NMR, 600 MHz in $CDCl_3$

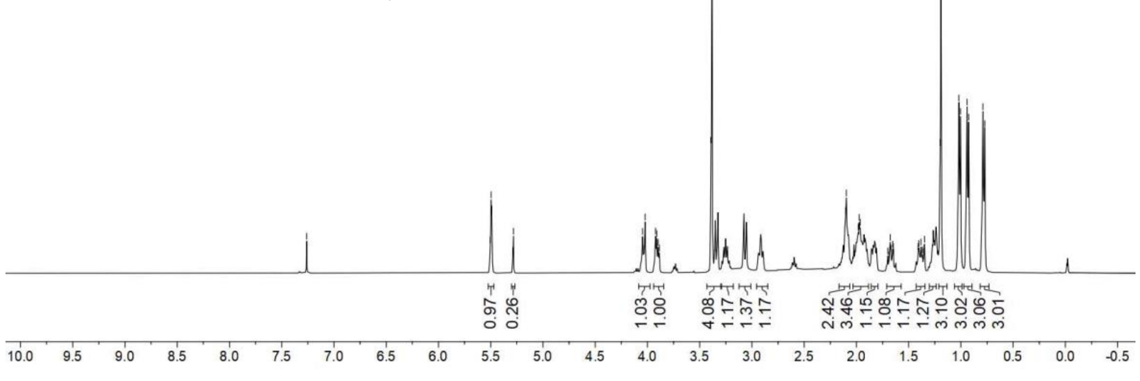


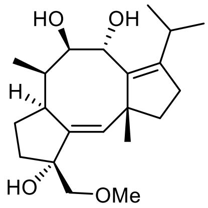


Cotylenol (**3**)
$^{13}$C NMR, 150 MHz in $CDCl_3$

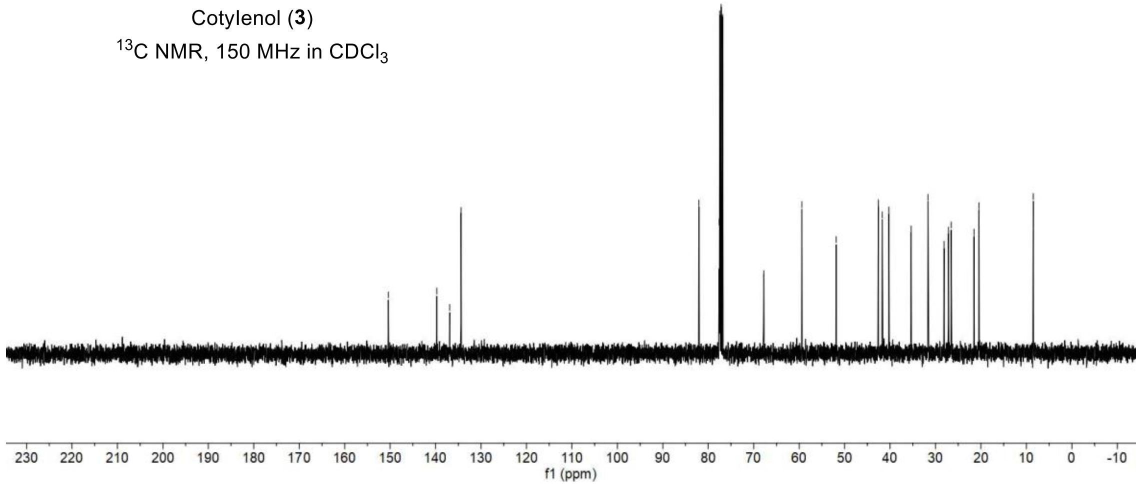

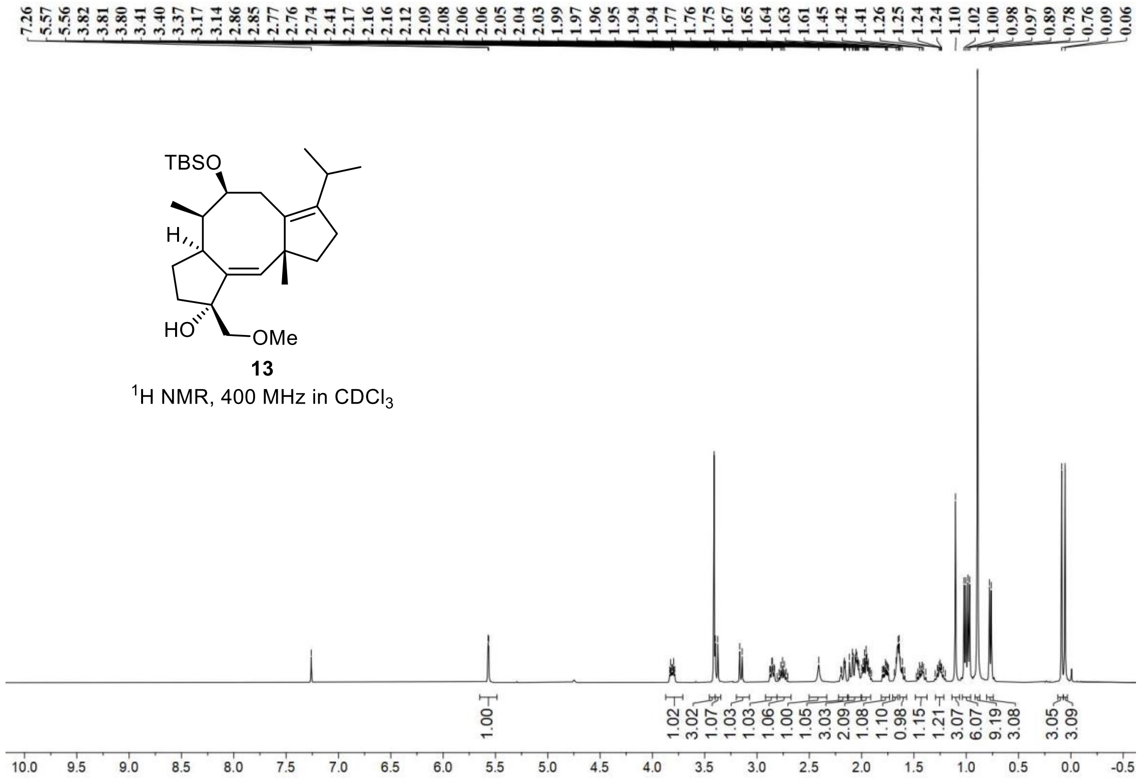
TBSO
H
HO
OMe
13
$^{1}H$ NMR, 400 MHz in $CDCl_3$

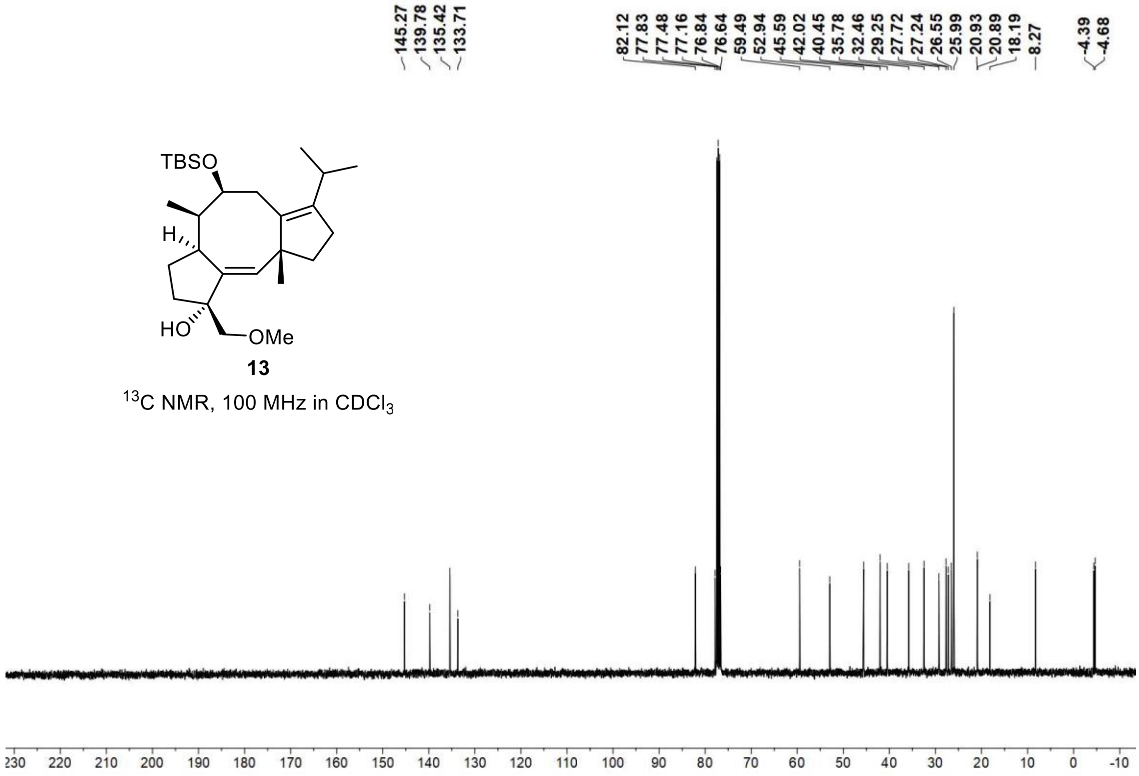
TBSO
H
HO
OMe
13
$^{13}C$ NMR, 100 MHz in $CDCl_3$

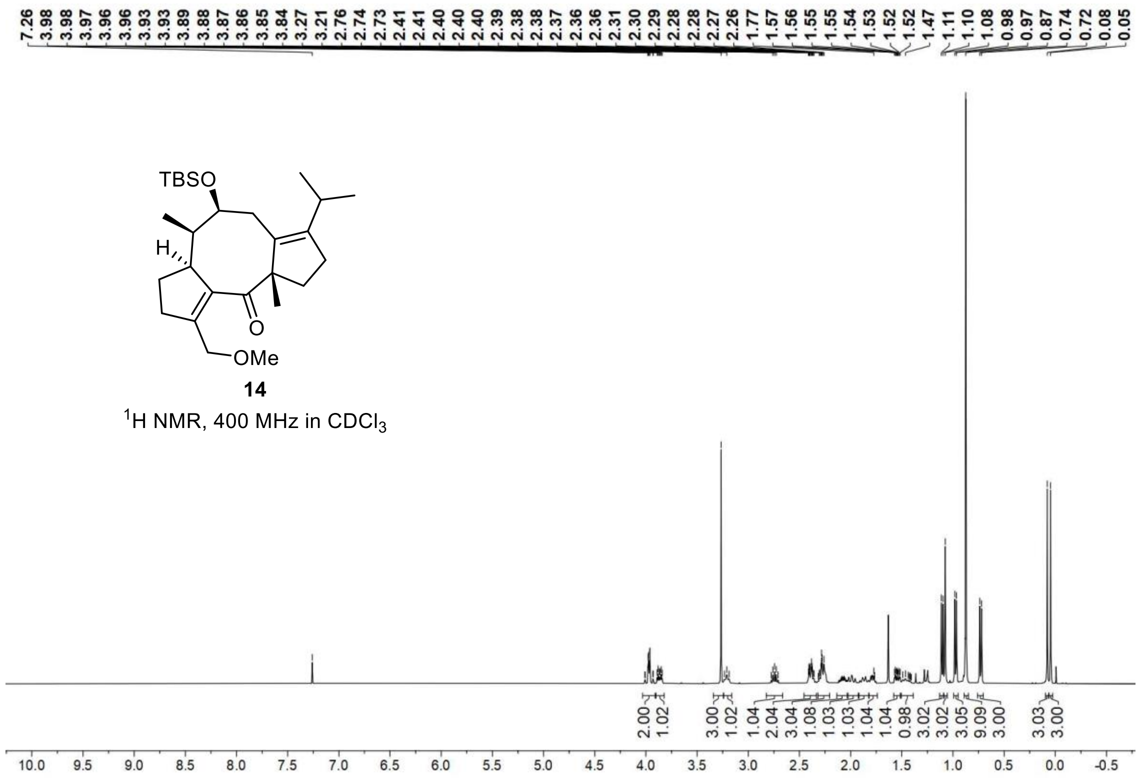


**14**

$^{1}H$ NMR, 400 MHz in $CDCl_3$

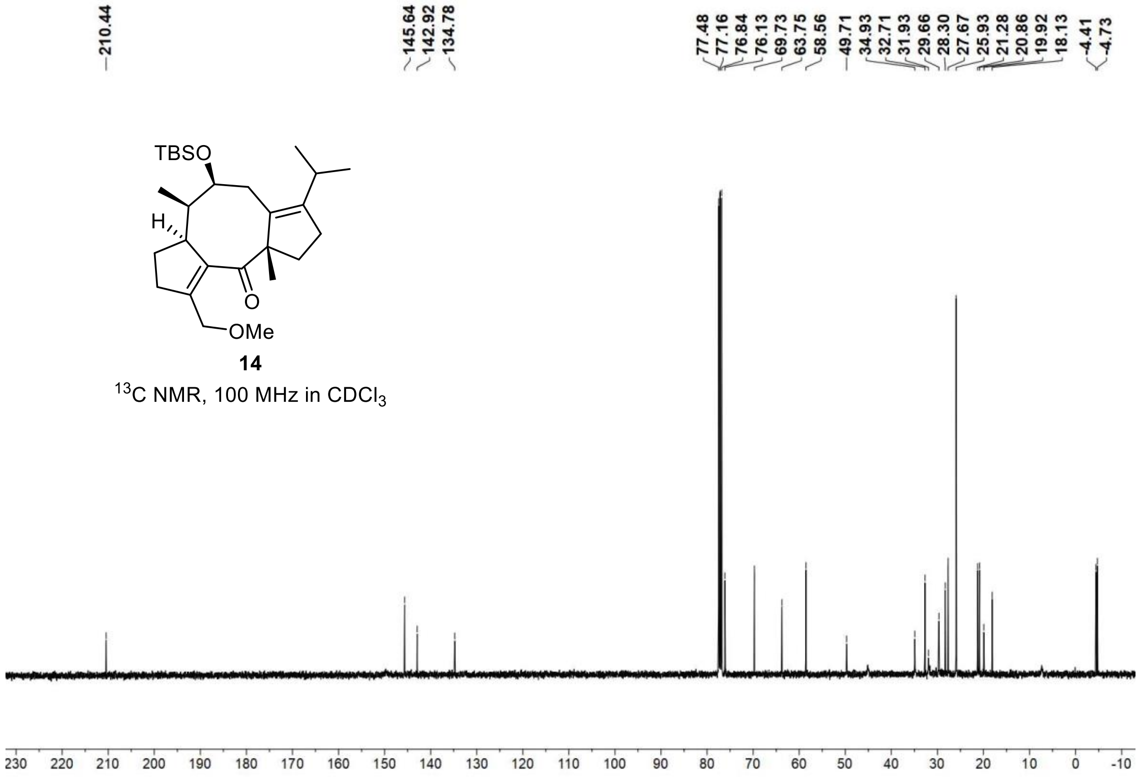


**14**

$^{13}C$ NMR, 100 MHz in $CDCl_3$

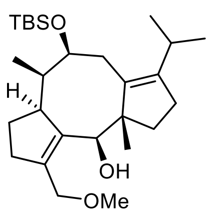


**15**

$^{1}H$ NMR, 400 MHz in $CDCl_3$

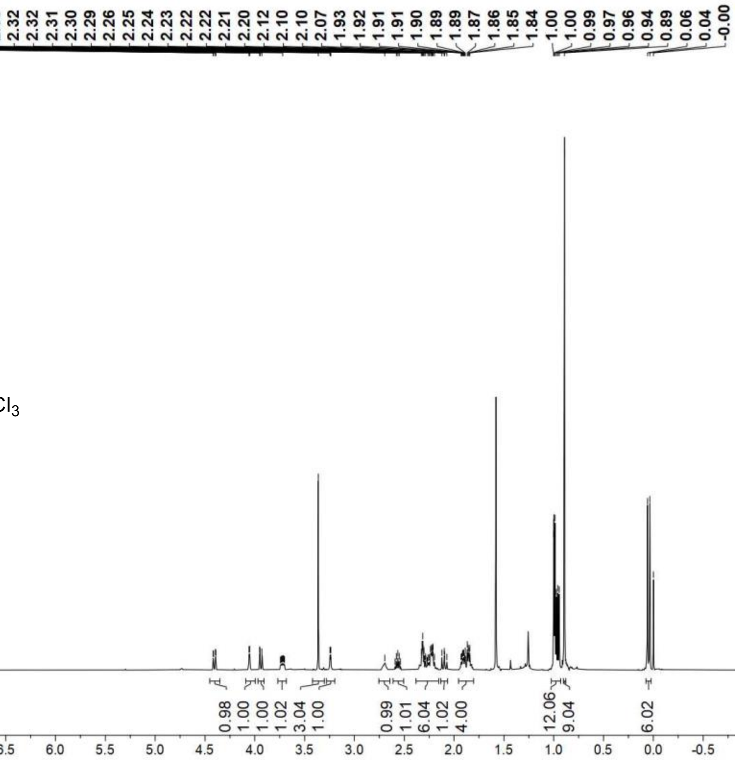


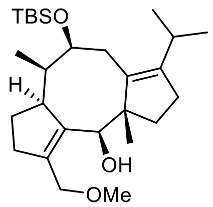


**15**

$^{13}C$ NMR, 100 MHz in $CDCl_3$

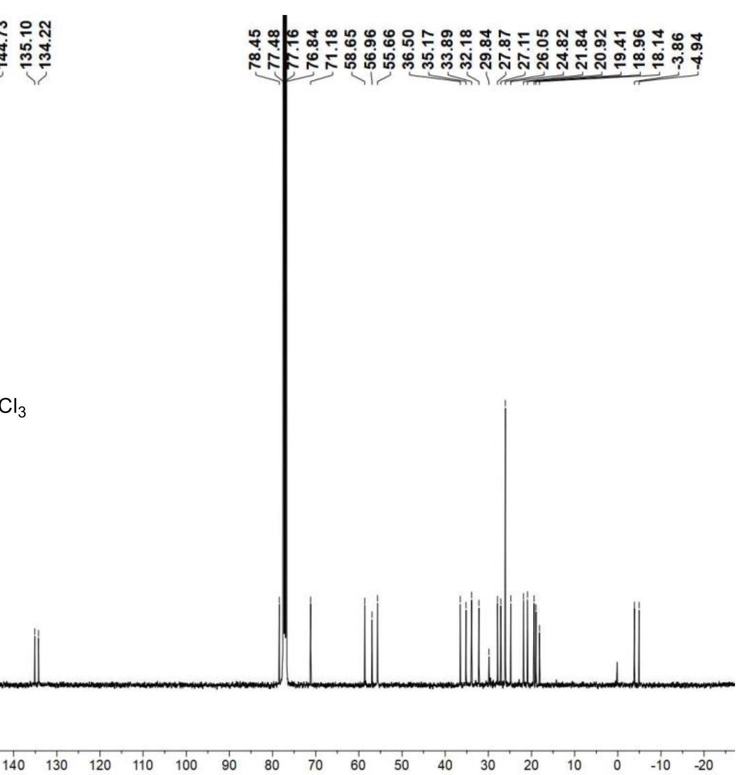

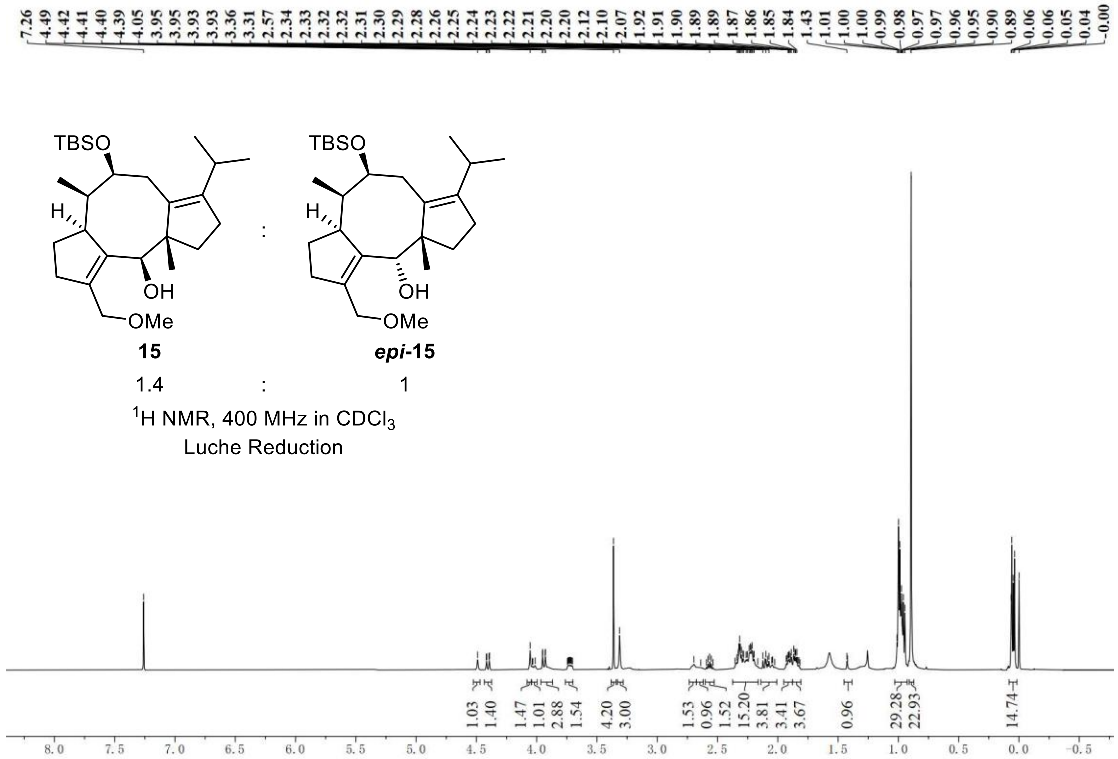
7.26 4.49 4.42 4.41 4.40 4.39 4.05 3.95 3.95 3.93 3.93 3.36 3.31 2.57 2.34 2.33 2.32 2.32 2.31 2.30 2.29 2.28 2.26 2.25 2.24 2.23 2.22 2.21 2.20 2.20 2.12 2.10 2.07 1.92 1.91 1.90 1.89 1.89 1.87 1.86 1.85 1.84 1.43 1.01 1.00 1.00 0.99 0.98 0.97 0.97 0.96 0.95 0.90 0.89 0.06 0.06 0.05 0.04 -0.00
TBSO
H
OH
OMe
15
epi-15
1.4 : 1
$^{1}$H NMR, 400 MHz in $CDCl_3$
Luche Reduction
1.03 1.40 1.47 1.01 2.88 1.54 4.20 3.00 1.53 0.96 1.52 15.20 3.81 3.41 3.67 0.96 29.28 22.93 14.74
8.0 7.5 7.0 6.5 6.0 5.5 5.0 4.5 4.0 3.5 3.0 2.5 2.0 1.5 1.0 0.5 0.0 -0.5

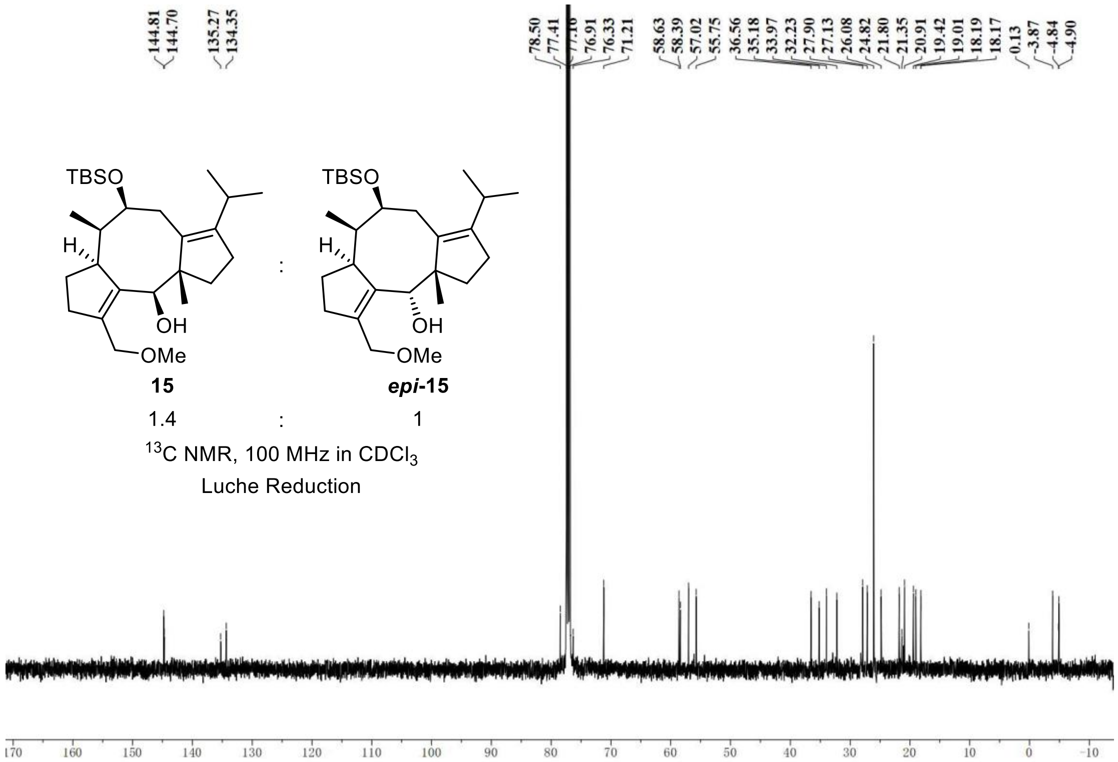
144.81 144.70 135.27 134.35 78.50 77.41 77.16 76.91 76.33 71.21 58.63 58.39 57.02 55.75 36.56 35.18 33.97 32.23 27.90 27.13 26.08 24.82 21.80 21.35 20.91 19.42 19.01 18.19 18.17 0.13 -3.87 -4.84 -4.90
TBSO
H
OH
OMe
15
epi-15
1.4 : 1
$^{13}$C NMR, 100 MHz in $CDCl_3$
Luche Reduction
170 160 150 140 130 120 110 100 90 80 70 60 50 40 30 20 10 0 -10

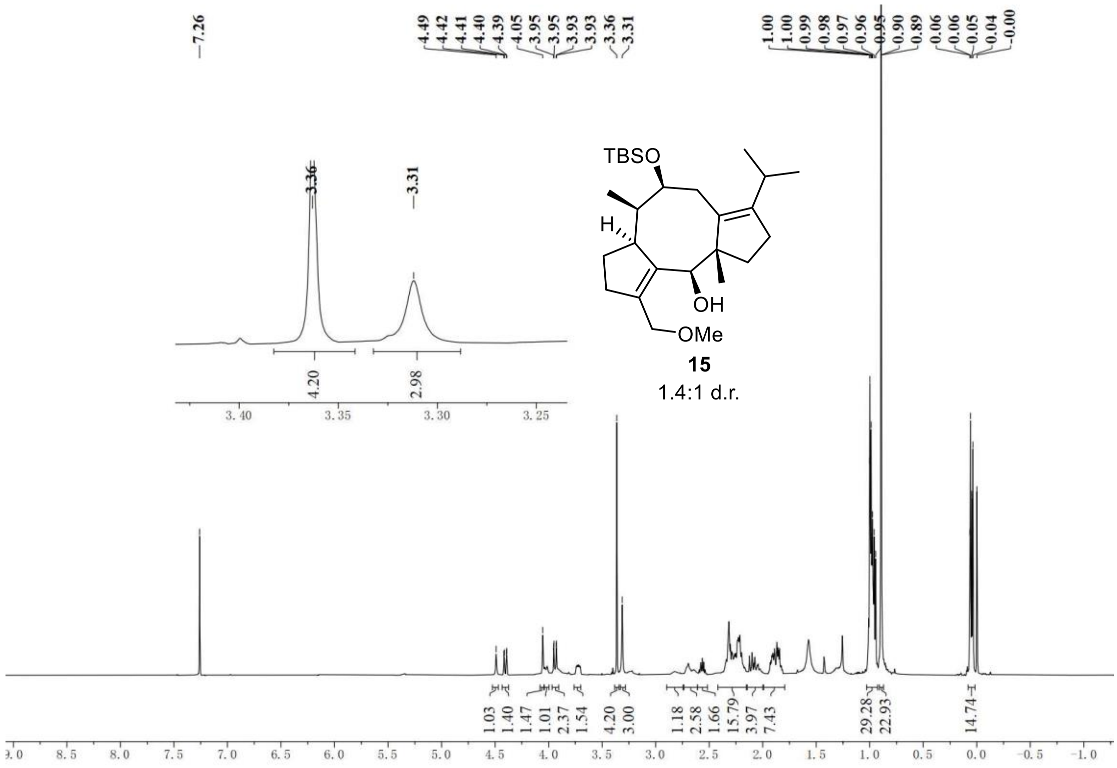


$^{1}$H NMR Spectrum of **15**, 1:1 d.r. (400 MHz, $CDCl_3$), by Luche reduction

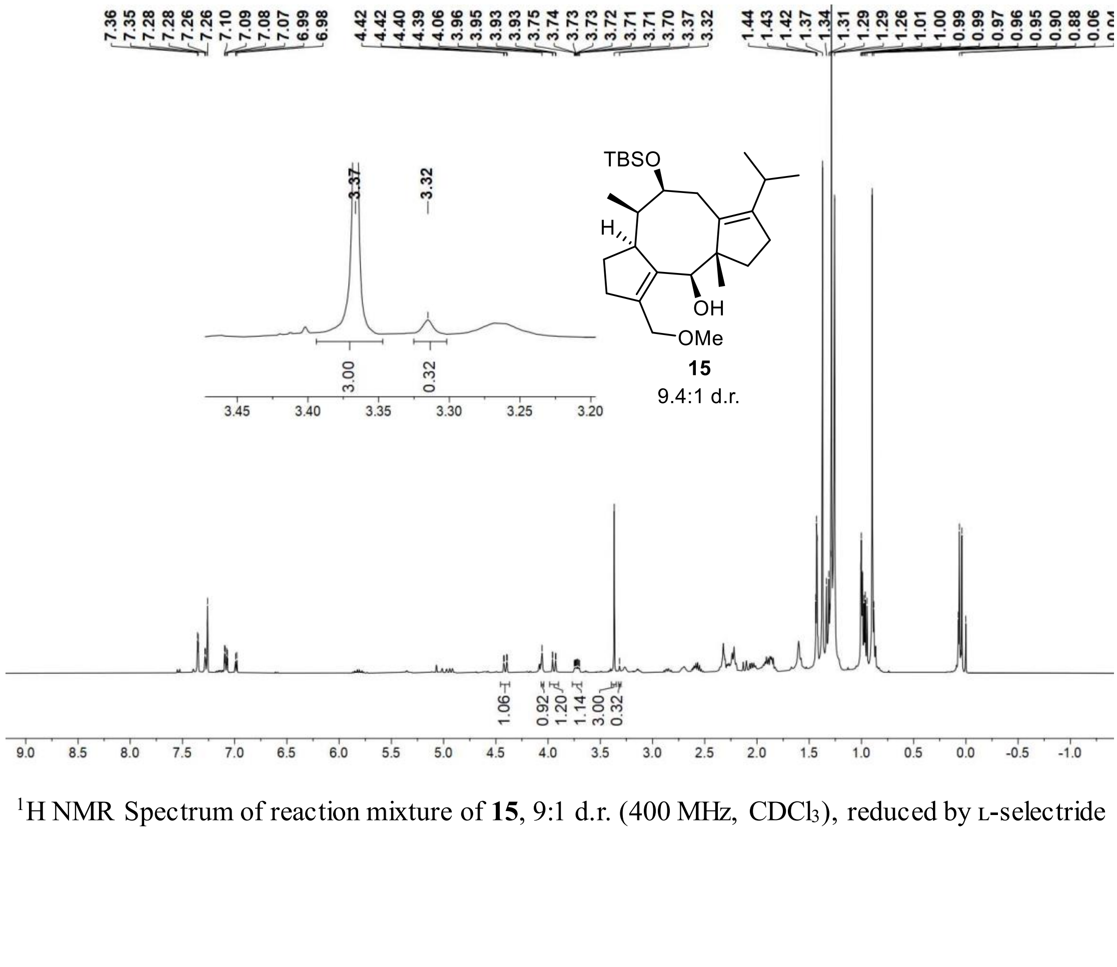


$^{1}$H NMR Spectrum of reaction mixture of **15**, 9:1 d.r. (400 MHz, $CDCl_3$), reduced by L-selectride

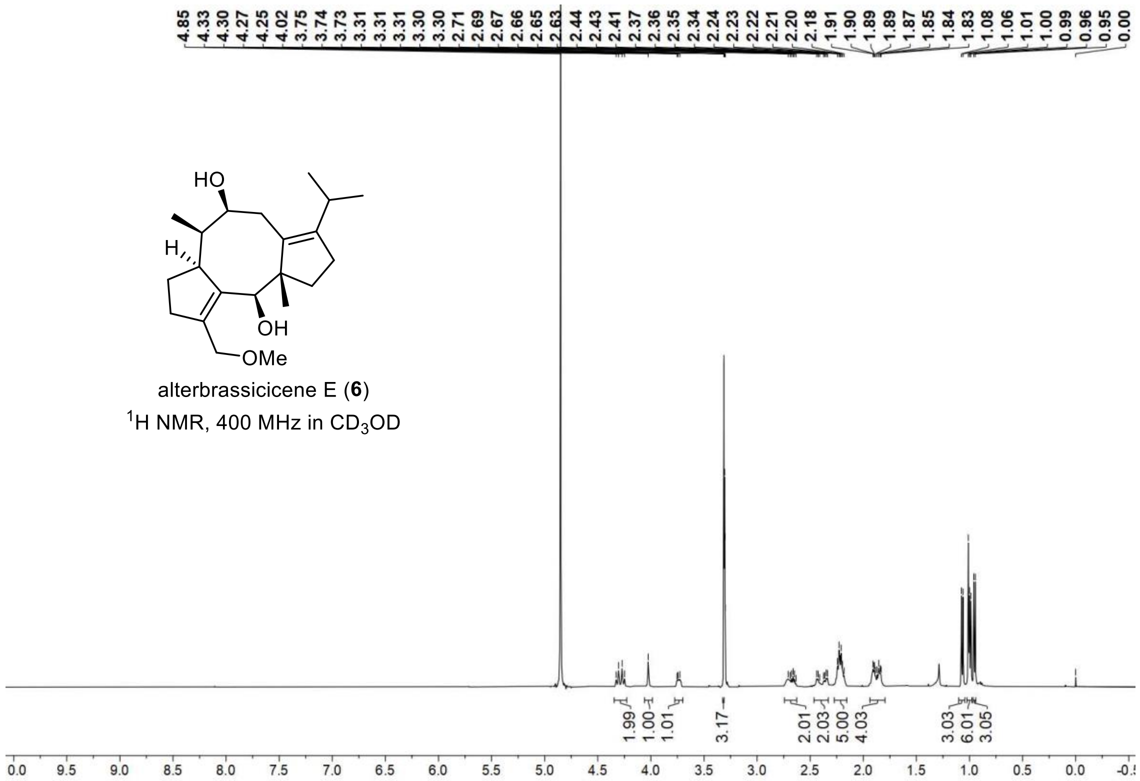


alterbrassicicene E (**6**)
$^{1}$H NMR, 400 MHz in $CD_3OD$

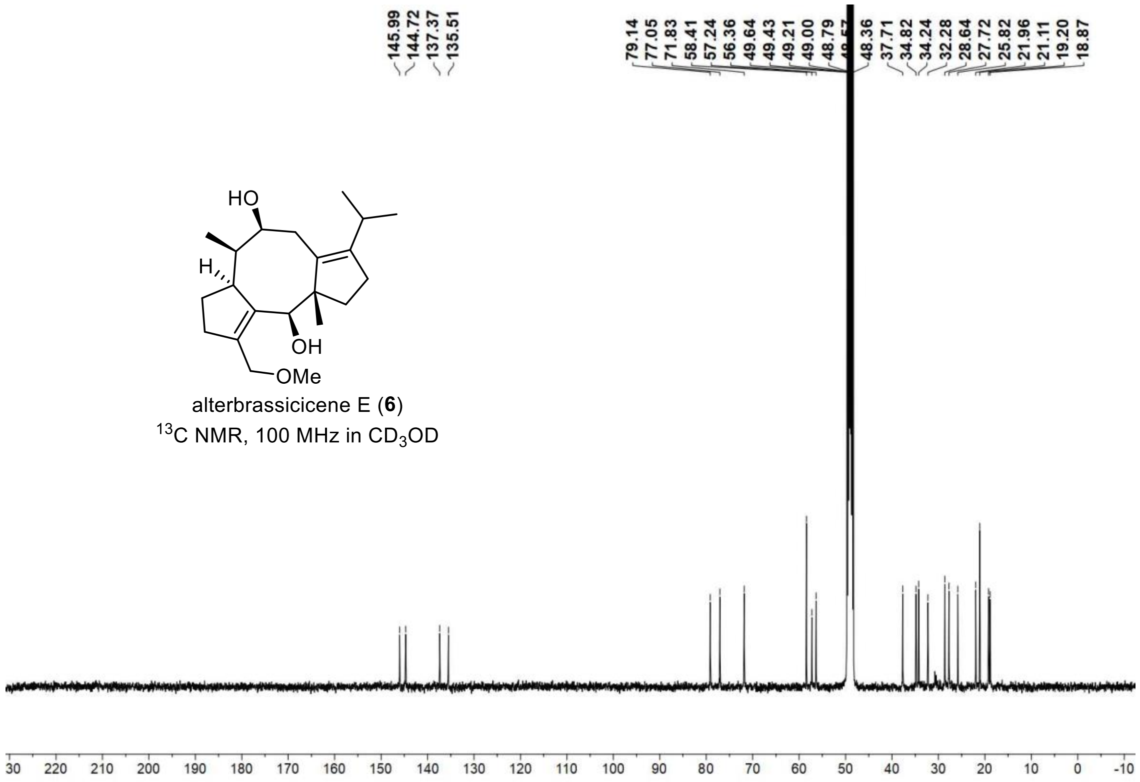


alterbrassicicene E (**6**)
$^{13}$C NMR, 100 MHz in $CD_3OD$

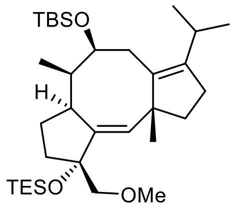


**16**

$^1$H NMR, 400 MHz in $CDCl_3$

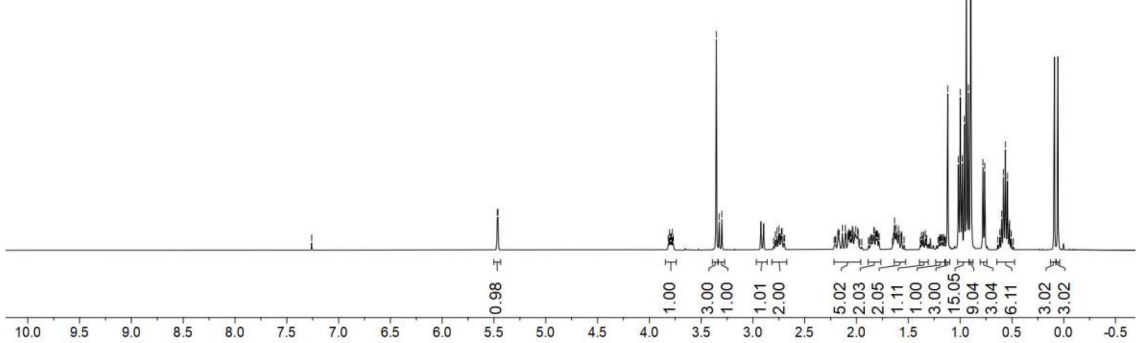


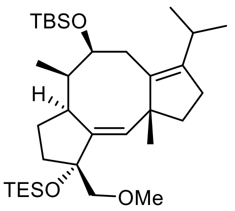


**16**

$^{13}$C NMR, 100 MHz in $CDCl_3$

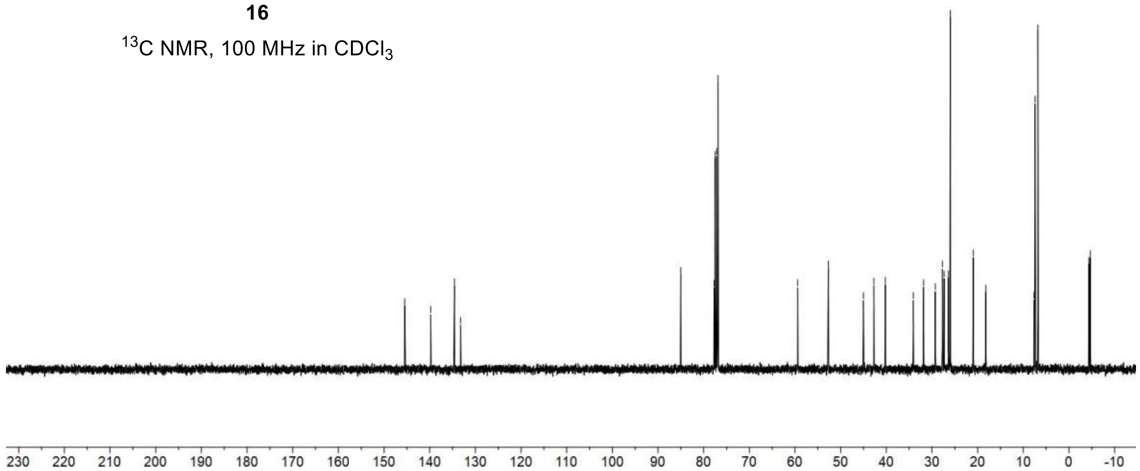

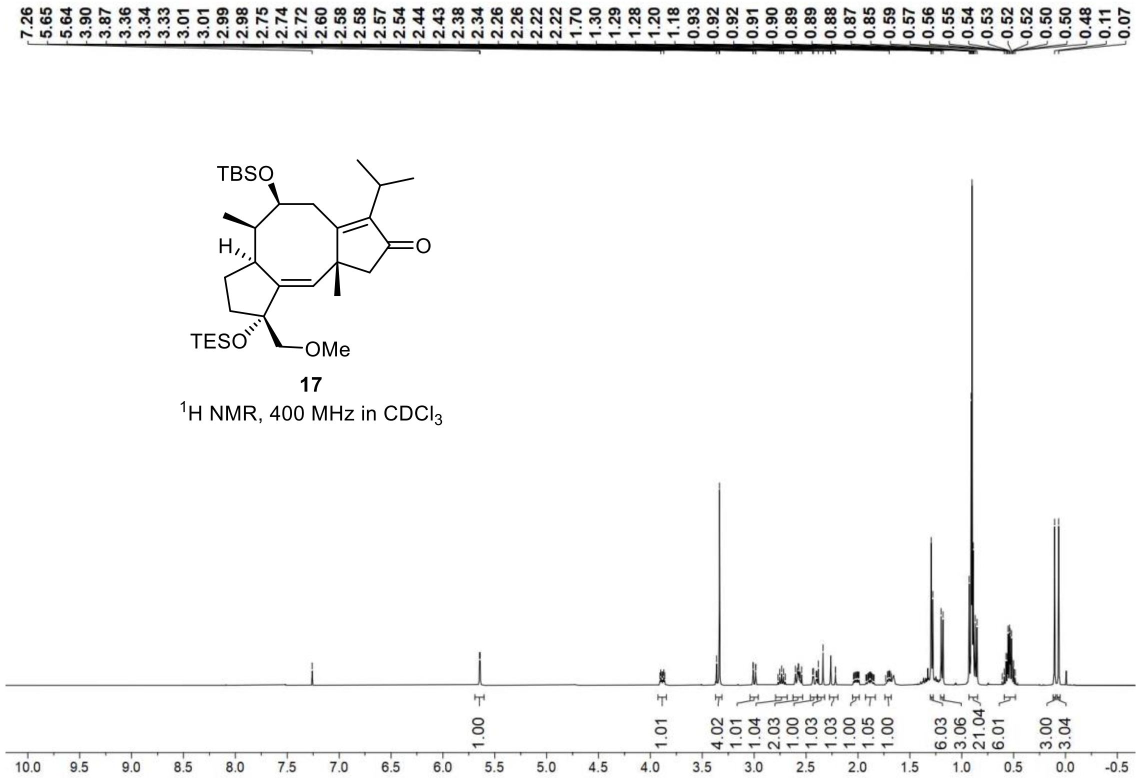

**17**

$^{1}$H NMR, 400 MHz in $CDCl_3$

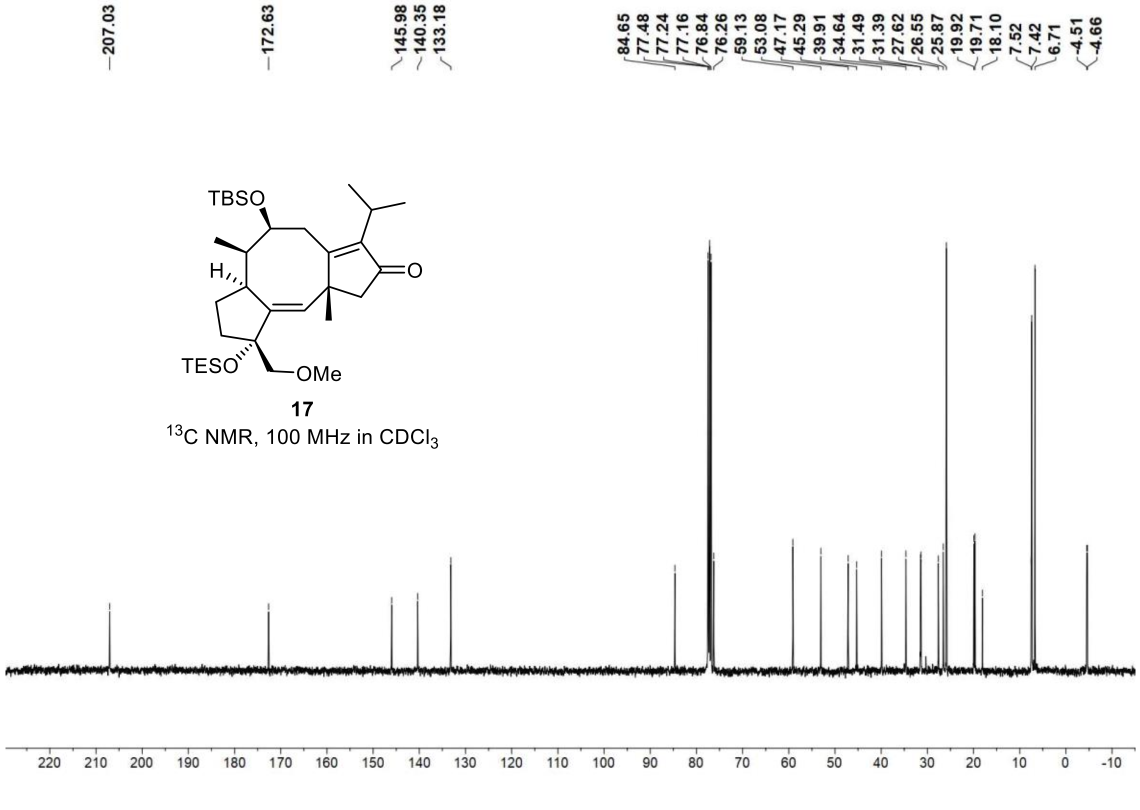

**17**

$^{13}$C NMR, 100 MHz in $CDCl_3$

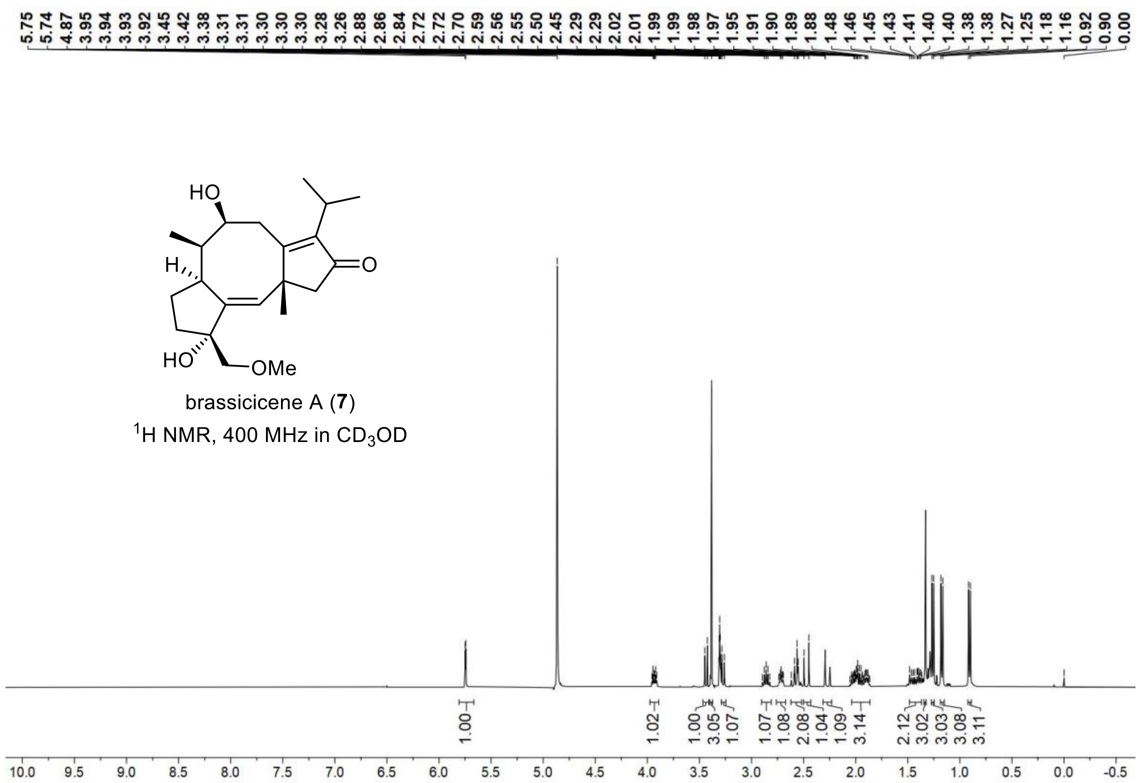
5.75 5.74 4.87 3.95 3.94 3.93 3.92 3.45 3.42 3.38 3.31 3.31 3.30 3.30 3.30 3.28 3.26 2.88 2.86 2.84 2.72 2.72 2.70 2.59 2.56 2.55 2.50 2.45 2.29 2.29 2.02 2.01 1.99 1.99 1.98 1.97 1.95 1.91 1.90 1.89 1.88 1.48 1.46 1.45 1.43 1.41 1.40 1.40 1.38 1.27 1.25 1.18 1.16 0.92 0.90 0.00
HO
H
O
HO
OMe
brassicicene A (7)
1H NMR, 400 MHz in CD3OD
1.00 1.02 1.00 3.05 1.07 1.07 1.08 2.08 1.04 1.09 3.14 2.12 3.02 3.03 3.08 3.11
10.0 9.5 9.0 8.5 8.0 7.5 7.0 6.5 6.0 5.5 5.0 4.5 4.0 3.5 3.0 2.5 2.0 1.5 1.0 0.5 0.0 -0.5

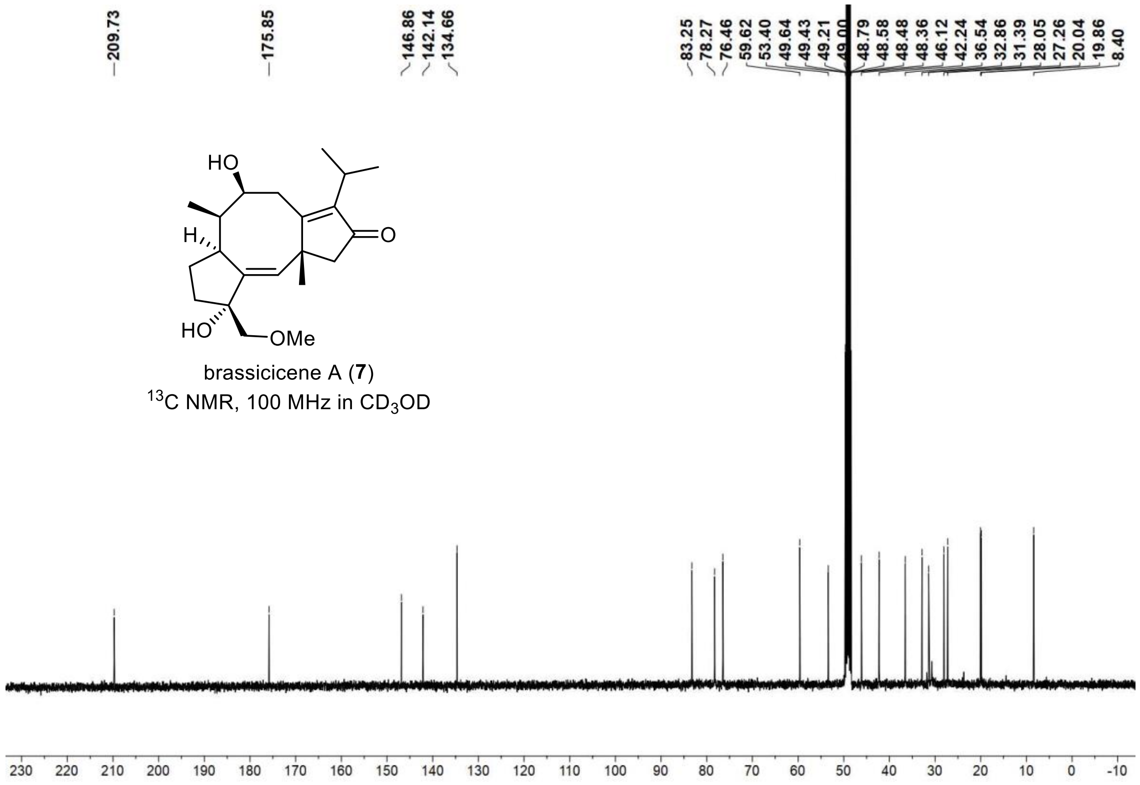
209.73
175.85
146.86 142.14 134.66
83.25 78.27 76.46 59.62 53.40 49.64 49.43 49.21 49.00 48.79 48.58 48.48 48.36 46.12 42.24 36.54 32.86 31.39 28.05 27.26 20.04 19.86 8.40
HO
H
O
HO
OMe
brassicicene A (7)
13C NMR, 100 MHz in CD3OD
230 220 210 200 190 180 170 160 150 140 130 120 110 100 90 80 70 60 50 40 30 20 10 0 -10

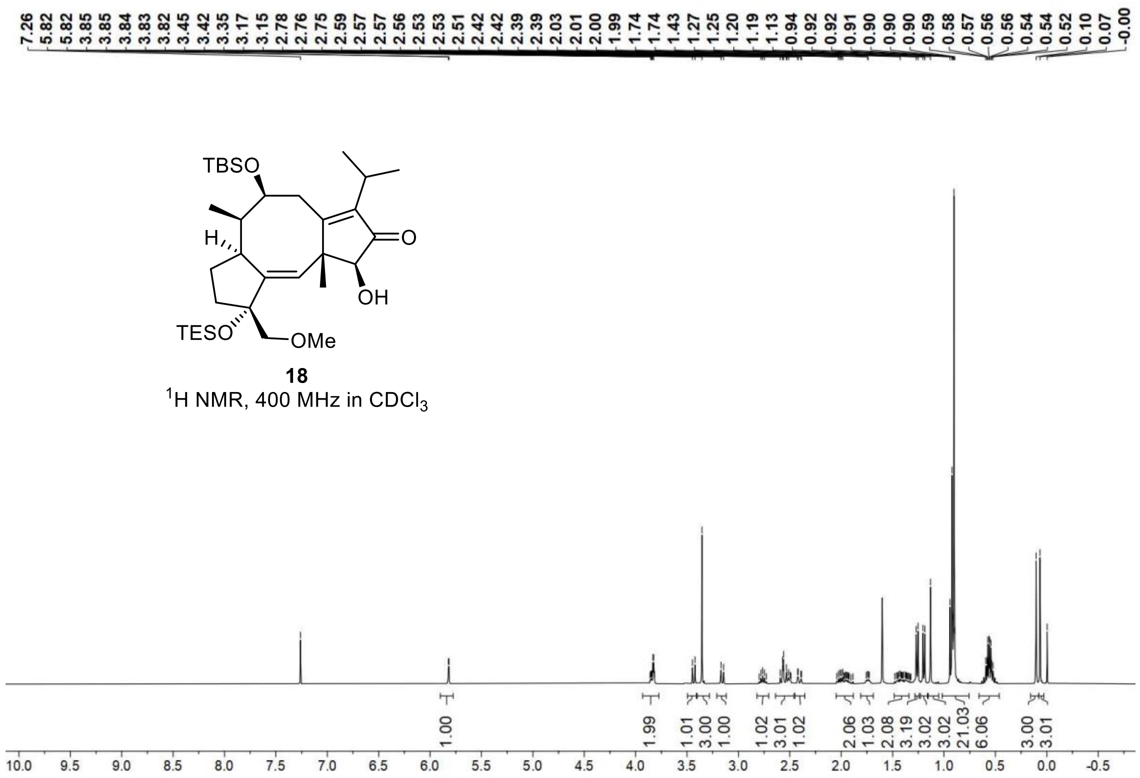
7.26 5.82 5.82 3.85 3.85 3.84 3.83 3.82 3.45 3.42 3.35 3.17 3.15 2.78 2.76 2.75 2.59 2.57 2.57 2.56 2.53 2.53 2.51 2.42 2.42 2.39 2.39 2.03 2.01 2.00 1.99 1.74 1.74 1.43 1.27 1.25 1.20 1.19 1.13 0.94 0.92 0.91 0.90 0.90 0.90 0.59 0.58 0.57 0.56 0.56 0.54 0.54 0.52 0.10 0.07 -0.00
TBSO
H
O
OH
TESO
OMe
18
1H NMR, 400 MHz in CDCl3
1.00 1.99 1.01 3.00 1.00 1.02 3.01 1.02 2.06 1.03 2.08 3.19 3.02 3.02 21.03 6.06 3.00 3.01
10.0 9.5 9.0 8.5 8.0 7.5 7.0 6.5 6.0 5.5 5.0 4.5 4.0 3.5 3.0 2.5 2.0 1.5 1.0 0.5 0.0 -0.5

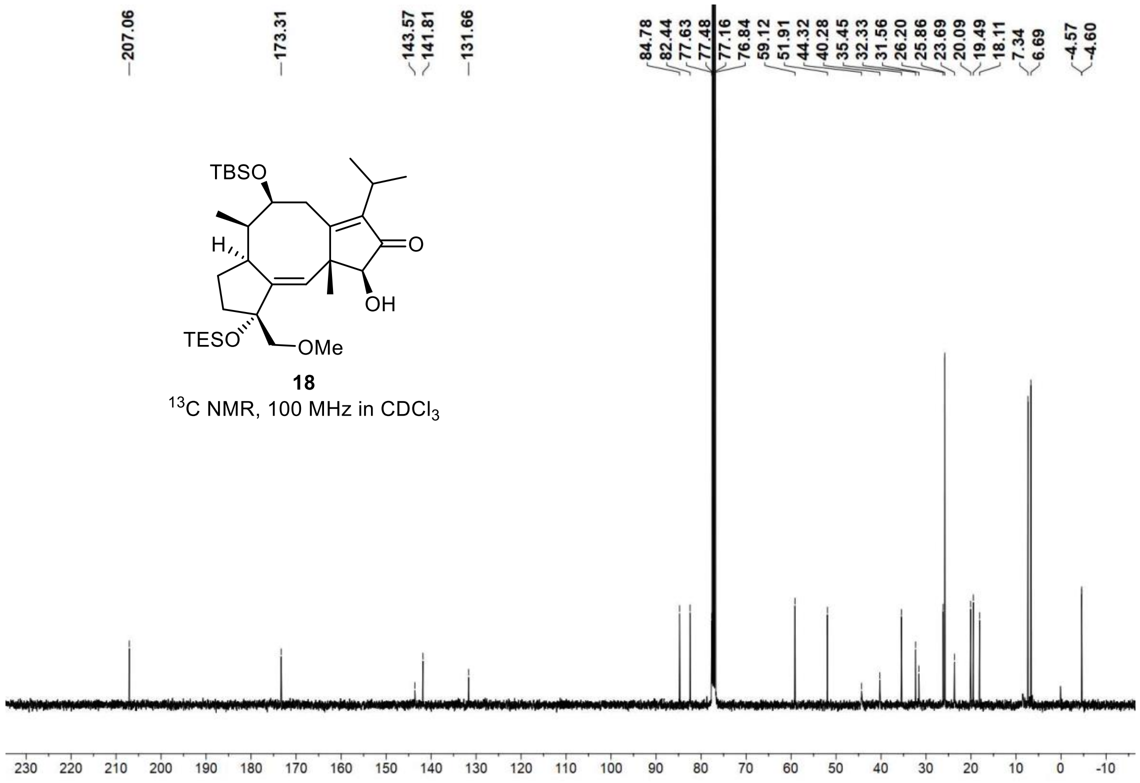
207.06 173.31 143.57 141.81 131.66 84.78 82.44 77.63 77.48 77.16 76.84 59.12 51.91 44.32 40.28 35.45 32.33 31.56 26.20 25.86 23.69 20.09 19.49 18.11 7.34 6.69 -4.57 -4.60
TBSO
H
O
OH
TESO
OMe
18
13C NMR, 100 MHz in CDCl3
230 220 210 200 190 180 170 160 150 140 130 120 110 100 90 80 70 60 50 40 30 20 10 0 -10

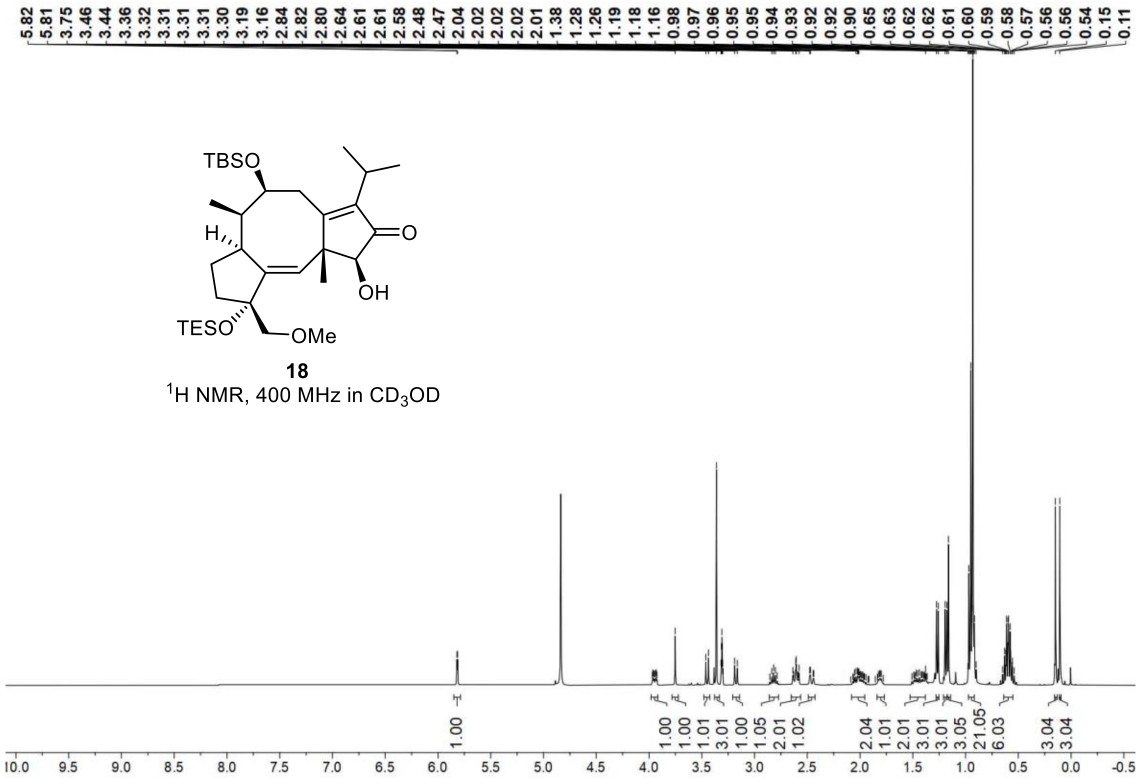


**18**

$^{1}$H NMR, 400 MHz in $CD_3OD$

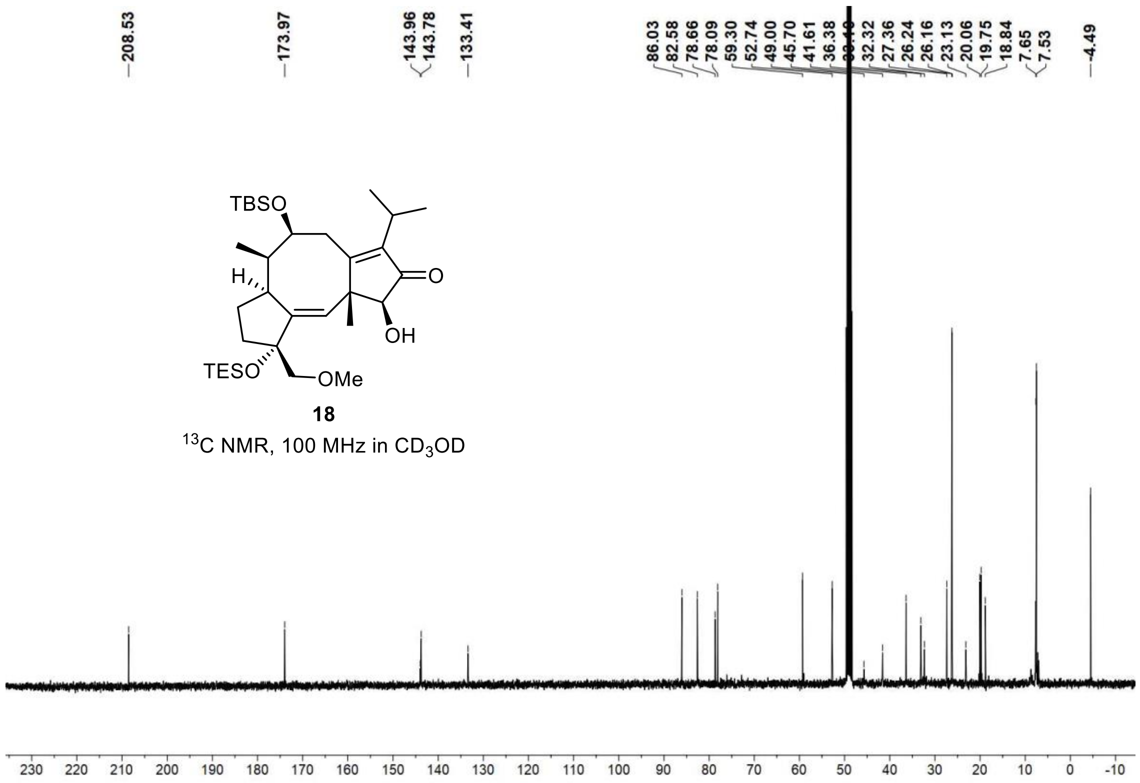


**18**

$^{13}$C NMR, 100 MHz in $CD_3OD$

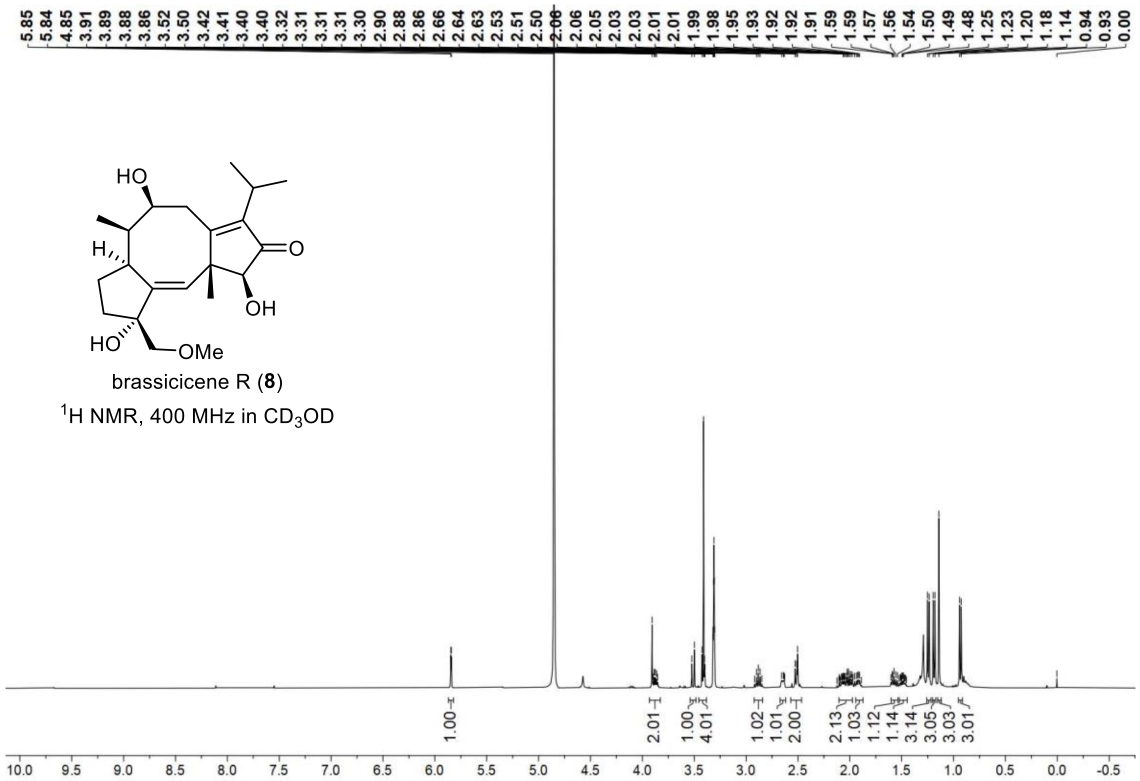
5.85 5.84 4.85 3.91 3.89 3.88 3.86 3.52 3.50 3.42 3.41 3.40 3.40 3.32 3.31 3.31 3.31 3.30 2.90 2.88 2.86 2.66 2.64 2.63 2.53 2.51 2.50 2.08 2.06 2.05 2.03 2.03 2.01 2.01 1.99 1.98 1.95 1.93 1.92 1.92 1.91 1.59 1.59 1.57 1.56 1.54 1.50 1.49 1.48 1.25 1.23 1.20 1.18 1.14 0.94 0.93 0.00
HO
H
O
OH
HO
OMe
brassicicene R (8)
$^1$H NMR, 400 MHz in $CD_3OD$
1.00 2.01 1.00 4.01 1.02 1.01 2.00 2.13 1.03 1.12 1.14 3.14 3.05 3.03 3.01
10.0 9.5 9.0 8.5 8.0 7.5 7.0 6.5 6.0 5.5 5.0 4.5 4.0 3.5 3.0 2.5 2.0 1.5 1.0 0.5 0.0 -0.5

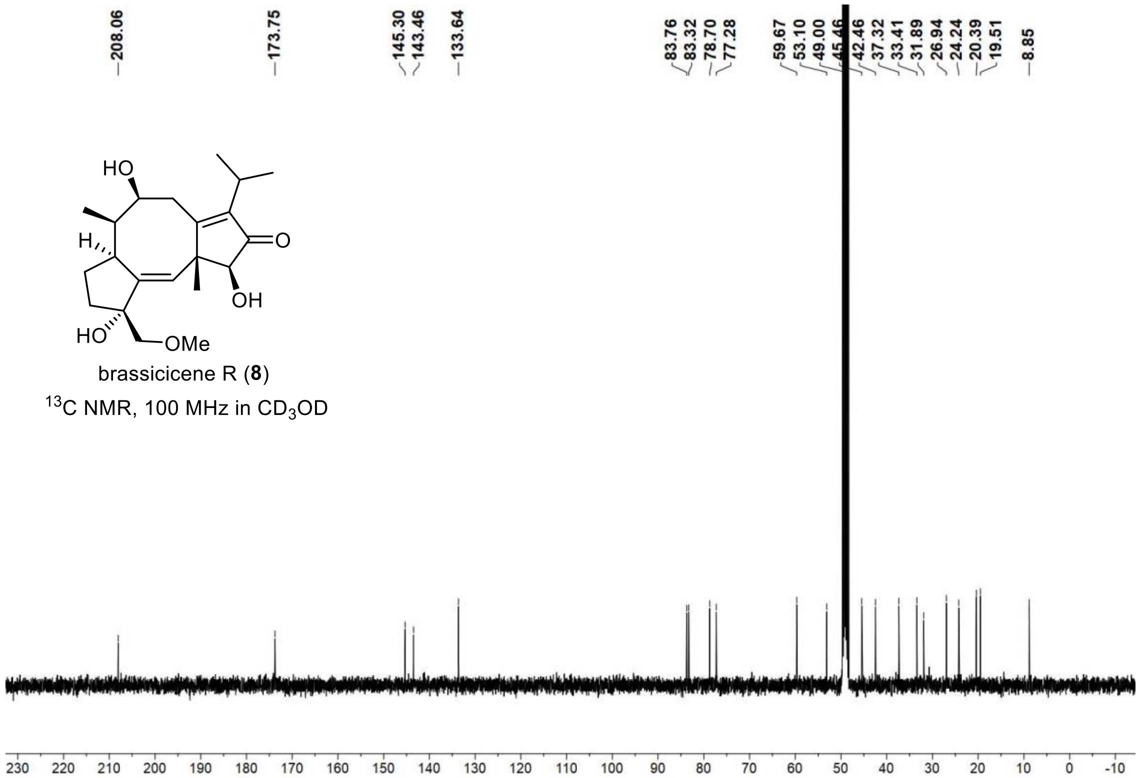
208.06 173.75 145.30 143.46 133.64 83.76 83.32 78.70 77.28 59.67 53.10 49.00 45.46 42.46 37.32 33.41 31.89 26.94 24.24 20.39 19.51 8.85
HO
H
O
OH
HO
OMe
brassicicene R (8)
$^{13}$C NMR, 100 MHz in $CD_3OD$
230 220 210 200 190 180 170 160 150 140 130 120 110 100 90 80 70 60 50 40 30 20 10 0 -10

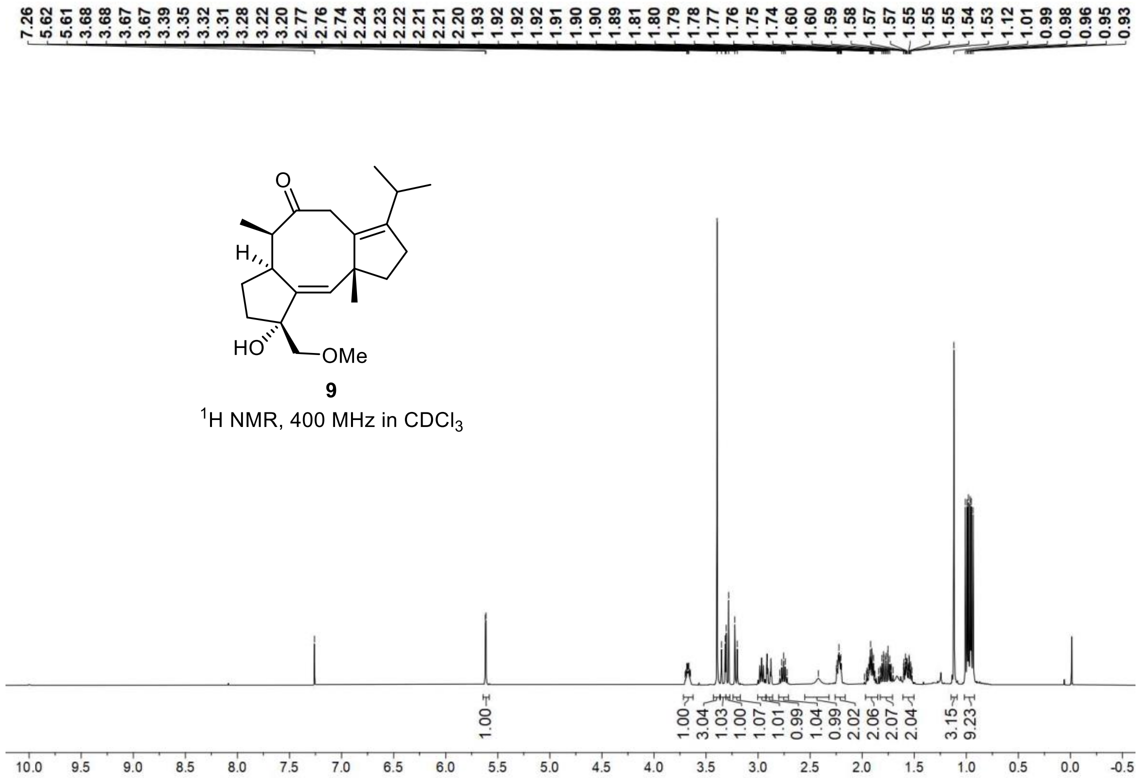
7.26 5.62 5.61 3.68 3.68 3.67 3.67 3.39 3.35 3.32 3.31 3.28 3.22 3.20 2.77 2.76 2.74 2.24 2.23 2.22 2.21 2.21 2.20 1.93 1.92 1.92 1.92 1.91 1.90 1.90 1.89 1.81 1.80 1.79 1.78 1.77 1.76 1.75 1.74 1.60 1.60 1.59 1.58 1.57 1.57 1.55 1.55 1.55 1.54 1.53 1.12 1.01 0.99 0.98 0.96 0.95 0.93
O
H
HO
OMe
9
1H NMR, 400 MHz in CDCl3
1.00 1.00 3.04 1.03 1.00 1.07 1.01 0.99 1.04 0.99 2.02 2.06 2.07 2.04 3.15 9.23
10.0 9.5 9.0 8.5 8.0 7.5 7.0 6.5 6.0 5.5 5.0 4.5 4.0 3.5 3.0 2.5 2.0 1.5 1.0 0.5 0.0 -0.5

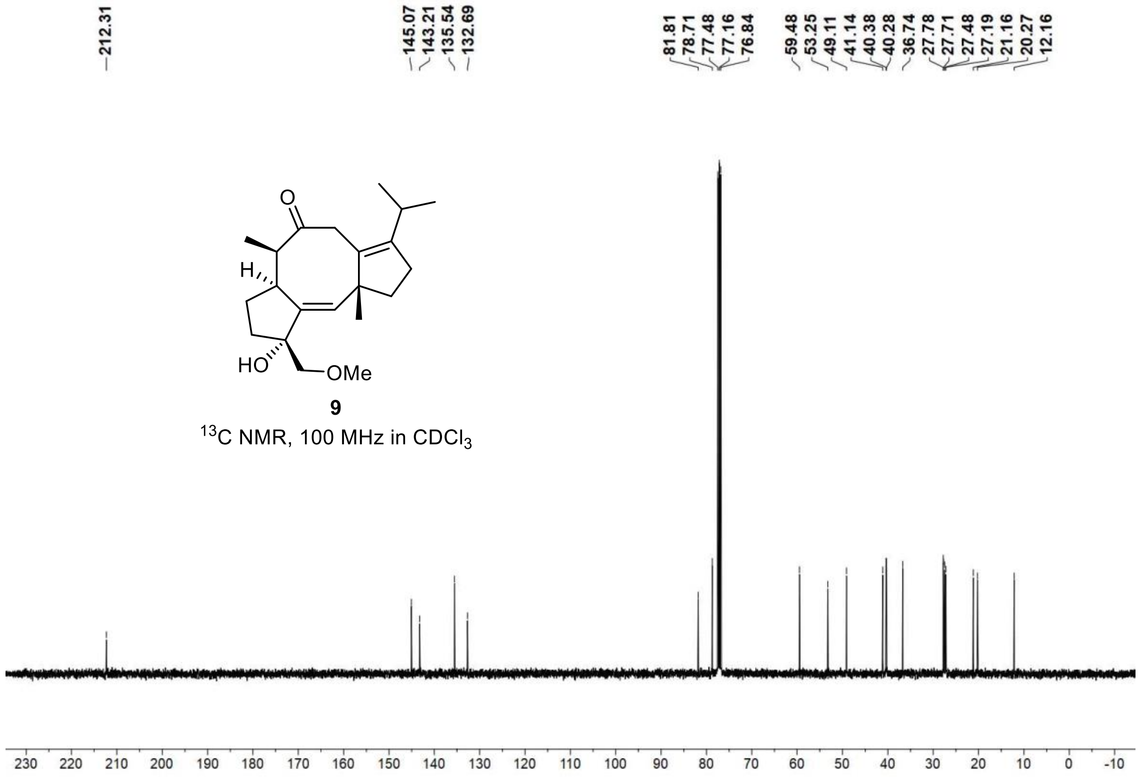
212.31
145.07 143.21 135.54 132.69
81.81 78.71 77.48 77.16 76.84
59.48 53.25 49.11 41.14 40.38 40.28 36.74 27.78 27.71 27.48 27.19 21.16 20.27 12.16
O
H
HO
OMe
9
13C NMR, 100 MHz in CDCl3
230 220 210 200 190 180 170 160 150 140 130 120 110 100 90 80 70 60 50 40 30 20 10 0 -10

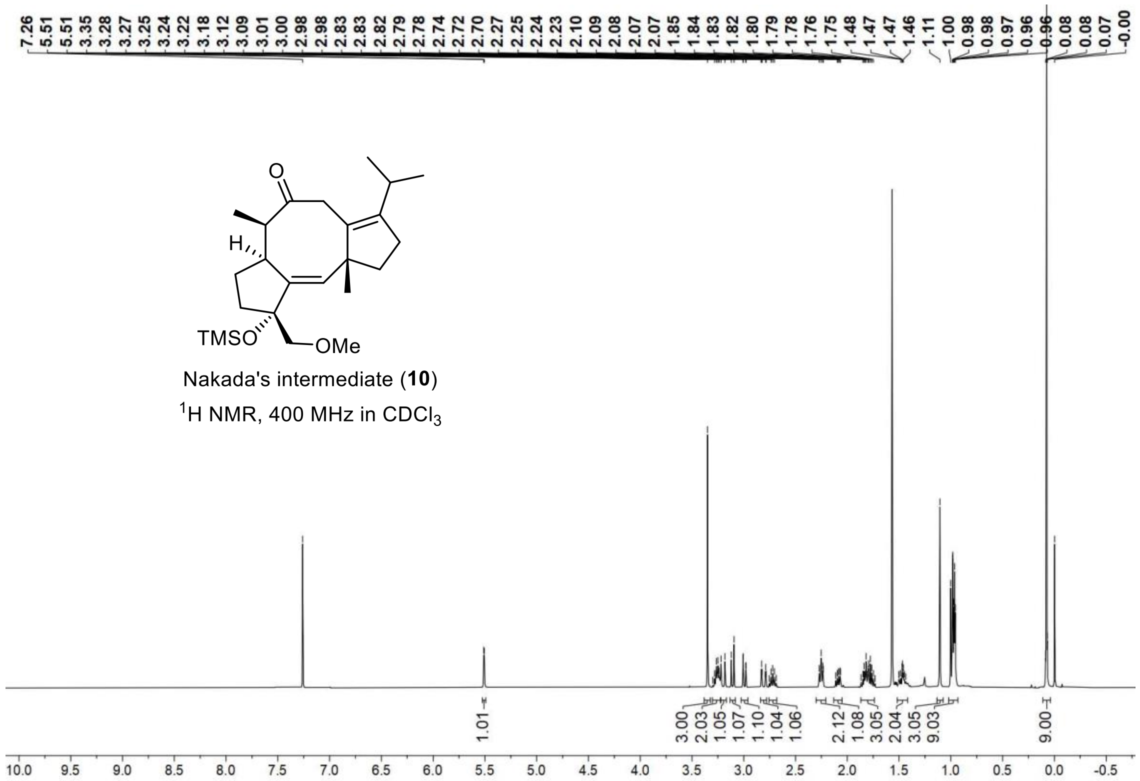
O
H
TMSO
OMe
Nakada's intermediate (10)
¹H NMR, 400 MHz in CDCl₃

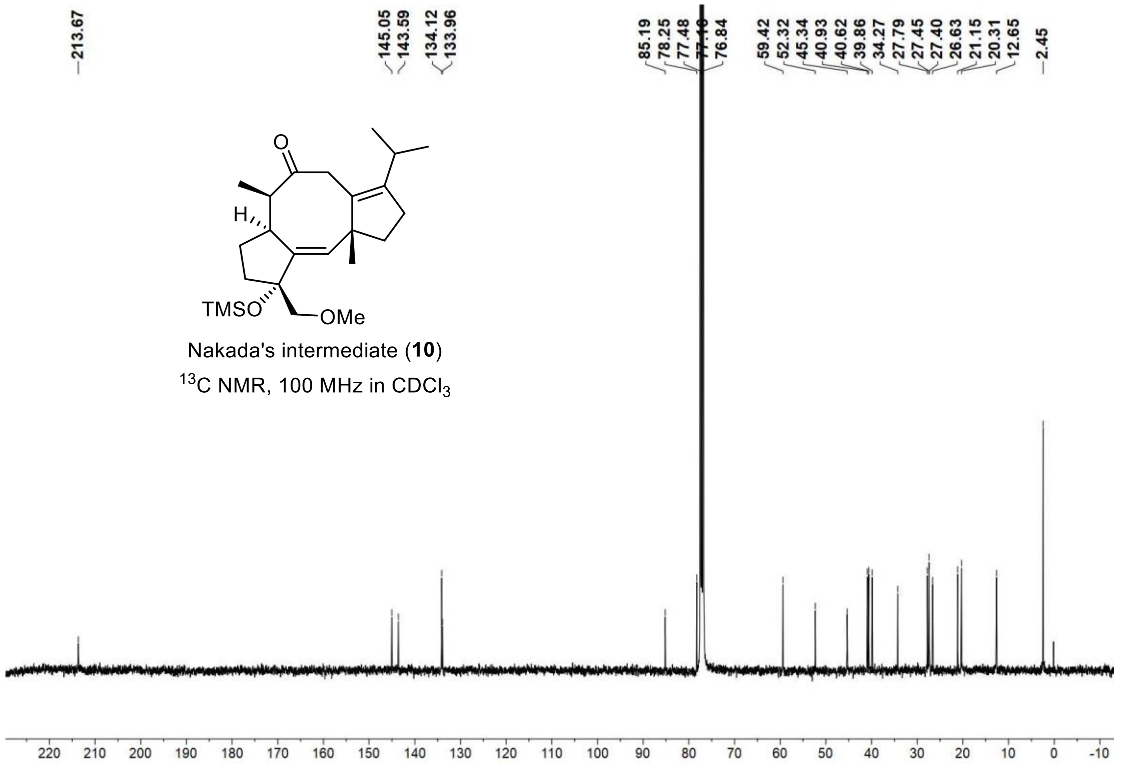
O
H
TMSO
OMe
Nakada's intermediate (10)
¹³C NMR, 100 MHz in CDCl₃
